# RSV replication modifies the XBP1s binding complex on the IRF1 upstream enhancer to potentiate the mucosal anti-viral response

**DOI:** 10.3389/fimmu.2023.1197356

**Published:** 2023-07-26

**Authors:** Dianhua Qiao, Xiaofang Xu, Yueqing Zhang, Jun Yang, Allan R. Brasier

**Affiliations:** ^1^ Department of Medicine, University of Wisconsin-Madison School of Medicine and Public Health (SMPH), Madison, WI, United States; ^2^ Department of Internal Medicine, University of Texas Medical Branch, Galveston, TX, United States; ^3^ Institute for Clinical and Translational Research (ICTR), University of Wisconsin-Madison, Madison, WI, United States

**Keywords:** inositol requiring enzyme (IRE1), X-box binding protein 1 (XBP1), interferon regulatory factor 1, innate immunity, Cleavage Under Targets and Release Using Nuclease (CUT&RUN)

## Abstract

**Introduction:**

The unfolded protein response (UPR) has emerged as an important signaling pathway mediating anti-viral defenses to Respiratory Syncytial Virus (RSV) infection. Earlier we found that RSV replication predominantly activates the evolutionarily conserved Inositol Requiring Enzyme 1α (IRE1α)-X-Box Binding Protein 1 spliced (XBP1s) arm of the Unfolded Protein Response (UPR) producing inflammation, metabolic adaptation and cellular plasticity, yet the mechanisms how the UPR potentiates inflammation are not well understood.

**Methods:**

To understand this process better, we examined the genomic response integrating RNA-seq and Cleavage Under Targets and Release Using Nuclease (CUT&RUN) analyses. These data were integrated with an RNA-seq analysis conducted on RSV-infected small airway cells ± an IRE1α RNAse inhibitor.

**Results:**

We identified RSV induced expression changes in ~3.2K genes; of these, 279 required IRE1α and were enriched in IL-10/cytokine signaling pathways. From this data set, we identify those genes directly under XBP1s control by CUT&RUN. Although XBP1s binds to ~4.2 K high-confidence genomic binding sites, surprisingly only a small subset of IL10/cytokine signaling genes are directly bound. We further apply CUT&RUN to find that RSV infection enhances XBP1s loading on 786 genomic sites enriched in AP1/Fra-1, RELA and SP1 binding sites. These control a subset of cytokine regulatory factor genes including IFN response factor 1 (IRF1), CSF2, NFKB1A and DUSP10. Focusing on the downstream role of IRF1, selective knockdown (KD) and overexpression experiments demonstrate IRF1 induction controls type I and -III interferon (IFN) and IFN-stimulated gene (ISG) expression, demonstrating that ISG are indirectly regulated by XBP1 through IRF1 transactivation. Examining the mechanism of IRF1 activation, we observe that XBP1s directly binds a 5’ enhancer sequence whose XBP1s loading is increased by RSV. The functional requirement for the enhancer is demonstrated by targeting a dCas9-KRAB silencer, reducing IRF1 activation. Chromatin immunoprecipitation shows that XBP1 is required, but not sufficient, for RSV-induced recruitment of activated phospho-Ser2 Pol II to the enhancer.

**Discussion:**

We conclude that XBP1s is a direct activator of a core subset of IFN and cytokine regulatory genes in response to RSV. Of these IRF1 is upstream of the type III IFN and ISG response. We find that RSV modulates the XBP1s binding complex on the IRF1 5’ enhancer whose activation is required for IRF1 expression. These findings provide novel insight into how the IRE1α-XBP1s pathway potentiates airway mucosal anti-viral responses.

## 1 Introduction

The host-restricted orthopneumovirus, respiratory syncytial virus (RSV), is an important etiological pathogen in human lower respiratory tract infections producing substantial acute- and long-term morbidity. As a highly infectious pathogen, RSV is transmitted via large droplet spread. In children less than five years of age, sequelae of RSV infections are the most common cause of pediatric hospitalization (1) and responsible for 1/3 of lower respiratory tract infections (LRTIs) globally (2). A large observational cohort study concluded that severe LRTIs were associated with a 2-fold increased risk of premature adult death from respiratory disease (3). Prior to the COVID pandemic, RSV was responsible for seasonal outbreaks of respiratory tract infections worldwide (4), but its mode of transmission is now changing (5).

Studies in naturally acquired (6) and experimentally induced (7) infections have shown that RSV infects epithelial cells derived from the upper and lower airways triggering an anti-viral response consisting of coordinate waves of cytokine (8, 9), IFN (10), and damage-associated pattern secretion (11). Although RSV replicates in diverse epithelial types, it is RSV replication in small bronchiolar epithelium that is pathogenic for severe disease (inflammation and remodeling) because of cell-type differences in anti-viral response. Here, secretoglobin (*Scg1a1*+)- expressing Club cell progenitors in the small bronchioles produce greater amounts of neutrophilic-, T helper 2 (Th2)-polarizing-, and mucogenic cytokine production (12, 13). In addition, genetic knockouts of innate signaling in the Scg1a1+ population substantially reduces RSV induced neutrophilia and airway obstruction (12, 13). This mechanism explains the observations that, in fatal cases of RSV LRTI, small bronchiolar epithelial giant cell formation, necrosis and mucus plugs produce small airway obstruction, ventilation-perfusion mismatching, and acute hypoxic respiratory failure (6).

The mechanisms of innate signaling response to RSV infection have been intensively studied. Replicating in the cytoplasm, RSV ribonucleoprotein formation induces cytoplasmic stress granule formation; these dynamic, membraneless organelles are closely associated with the ER membrane and are sites for viral transcription/assembly and the initiation of the innate immune response (14, 15). Here, RSV RNA, RNA-binding proteins, translationally stalled cellular mRNAs accumulate and interact with cytoplasmic anti-viral sensors [pattern recognition receptors (PRRs)]. Active RSV transcription produces cytoplasmic dsRNAs and 5’ phosphorylated RNAs that represent pathogen-associated molecular patterns that coordinately activate the cytoplasmic retinoic acid inducible gene-I (RIG-I) and the membrane Toll-like receptor 4 (TLR4) (10). These PRRs trigger activation of mitogen activated protein (MAP) kinases, TANK-Binding Kinase and IκB Kinase (IKK) signalsomes resulting in AP1, IRF3 and NFκB activation (16, 17).

The innate anti-viral response is closely linked to the unfolded protein response (UPR) in RSV infection (18, 19). In cells replicating RSV, a rapid influx of RSV-encoded glycoproteins accumulate in the ER lumen, activating ER sensors, including inositol requiring enzyme (IRE1)α endonuclease, PKR-like ER kinase and activating transcription factor 6α (ATF6α) (20). Of these pathways, the IRE1α-XBP1s pathway is the primary pathway responsible for activating hexosamine biosynthesis, epithelial plasticity and secretion of innate response proteins and is the focus of this study. RSV triggers IRE1α activation by the accumulation of viral glycoproteins in the ER lumen that dissociate the immunoglobulin-binding chaperone Heat Shock Protein Family A (Hsp70) Member 5/BiP from IRE1α (21), triggering its autophosphorylation and activation of RNase activity. The IRE1α RNase alternatively splices a 26 nt fragment from X-box-binding protein 1 (XBP1) mRNA, forming a potent transcription factor that binds to GC-rich promoters recruiting activated RNA Polymerase (19). In this process, IRE1α-XBP1s pathway activates the hexosamine biosynthesis, shifting glycolysis to uridine diphosphate N-acetylglucosamine production (19, 22), enabling protein N-glycosylation, resolving proteotoxic stress by enabling basement membrane remodeling and processing viral glycoproteins (22). In parallel, IRE1α-XBP1s signaling supports epithelial mesenchymal plasticity (EMP) through activation of mesenchymal transcription factors ZEB1 and SNAI1. EMP promotes airway remodeling through ECM production and stimulation of myofibroblast formation in subepithelial fibroblasts (23).

Less well understood is how the UPR potentiates inflammatory and anti-viral responses (24). Earlier work has shown that the IRE1α RNase activity enhances the production of dsRNA patterns from cleaved cellular mRNAs enhancing MAP, TBK1 and IKK signaling (25). Others have found that XBP1s potentiates anti-viral signaling in response to TLR3/4, and IFNβ induction in macrophages by inducing assembly of a downstream enhancer enriched in IRF3 and p300 (26, 27). However, genome profiling studies using Cleavage Under Targets and Release Using Nuclease (CUT&RUN) have not been able to identify this IFNβ enhancer in epithelial cells (28). It is known that XBP1s binds pleiotropic sequence motifs affecting gene regulatory networks influenced by cell-type and stimulus-dependent control (29, 30). Hence the mechanisms how IRE1-XBP1s activate anti-viral signaling in small airway epithelial cells are enigmatic.

Our preliminary work has observed that the IRE1α-XBP1s pathway of the UPR dramatically influences expression of anti-viral cytokines in RSV-infected small airway epithelial cells (19). Based on its pleotropic binding patterns, we hypothesize that XBP1s is repositioned in the genome by the effects of RSV infection to potentiate the anti-viral response. To investigate this hypothesis, we integrated RNA-seq studies with CUT&RUN genomic profiling in small airway epithelial cells in the absence or presence of RSV infection. Quite strikingly, RSV activates a robust anti-viral gene regulatory network; however, surprisingly, these genes lack identifiable XBP1s binding peaks. We further identify ~786 genes whose XBP1s binding is modulated by RSV signaling. These XBP1s binding regions are enriched in AP-1, RELA and SP1-binding sequences. Of these, computational inference predicts XBP1s directly controls a core of cytokine regulatory factors that include interferon regulatory factor 1 (IRF1), colony stimulated factor (CSF)2, NFκB Inhibitor Alpha (NFKBIA), and dual specificity phosphatase (DUSP)10 genes; these genes influence innate signaling through diverse mechanisms. Here we focus on the role of IRF1; using knockdown and expression approaches, we demonstrate that IRF1 controls a type I and III IFN stimulated gene (ISG) network. XBP1s binds to the IRF1 upstream enhancer, which we demonstrate is functionally involved in IRF1 expression using site-specific targeting of the Krüppel associated box (KRAB) silencer. XBP1s binding to the IRF1 enhancer mediates XBP1s transactivation of IRF1 by recruitment of activated phospho-Ser2 Pol II. These data provide further mechanistic understanding how the IRE1α-XBP1s potentiates innate anti-viral signaling.

## 2 Materials and methods

### 2.1 Epithelial cell culture and treatment

Immortalized primary human small airway epithelial cells hSAECs and type II transformed alveolar carcinoma (A549) cells were obtained from American Type Culture Collection (ATCC, Gaithersburg, MD, USA). hSAECs were grown in SAGM (Lonza) and A549 in DMEM/F12 (Gibco supplemented with 10% FBS) in a humidified 5% CO_2_ environment (19, 23). RSV Long strain was prepared by sucrose cushion ultracentrifugation and titered by methylcellulose plaque assay (19). hSAECs were infected for 24 h at a multiplicity of infection (MOI) of 1.0 prior to harvest. For pharmacological induction of the UPR, hSAECs were treated for indicated times with various standard concentrations of tunicamycin (TM, 0.5-1 μg/ml) or thapsigargin (Tg, 50-100 nM). The kinase-inhibiting RNase attenuator (KIRA)-8, a selective IRE1α RNase inhibitor, was directly added to the SAGM at a concentration of 10 μM (31). The reagent was from MedChemExpress (South Brunswick Township, NJ, USA).

### 2.2 RNA isolation and quantitative RT-PCR (Q-RT-PCR)

Total cellular RNA was isolated and subjected to DNase digestion (Qiagen). Complementary DNAs (cDNAs) were synthesized with SuperScript III First Strand cDNA Synthesis Kit (Thermo Scientific). Q-RT-PCR assays were performed using SYBR Green Master Mix (Bio-Rad) and gene-specific primers (**Table 1**). *IFIT1, IFITM1* and *PPIA* mRNAs were analyzed by Taqman primers, Thermo Hs03027069_s1, Hs00705137_s1 and Hs04194521_s1, respectively. Data are presented as fold change normalizing to internal control PPIA using the ΔΔCt method.

**Table 1:** Q-RT-PCR primers. Primer pairs used for SYBR Green Q-RT-PCR of mRNA expression.

Gene	Primers
*IRF1*	F: 5’-GAGGAGGTGAAAGACCAGAGCA-3’ R: 5’-TAGCATCTCGGCTGGACTTCGA-3’
*CSF2*	F: 5’-GGAGCATGTGAATGCCATCCAG-3’ R: 5’-CTGGAGGTCAAACATTTCTGAGAT-3’
*INHBA*	F: 5’-GGATGACATTGGAAGGAGGGCA-3’ R: 5’-ACTGACAGGTCACTGCCTTCCT-3’
*NFKBIA*	F: 5’-TCCACTCCATCCTGAAGGCTAC-3’ R: 5’-CAAGGACACCAAAAGCTCCACG-3’
*DUSP10*	F: 5’-CAGCCACTTCACATAGTCCTCG-3’ R: 5’-TGGAGGGAGTTGTCACAGAGGT-3’
*IL15*	F: 5’-AACAGAAGCCAACTGGGTGAATG-3’ R: 5’-CTCCAAGAGAAAGCACTTCATTGC-3’
*IFNL2*	F: 5’-TCGCTTCTGCTGAAGGACTGCA-3’ R: 5’-CCTCCAGAACCTTCAGCGTCAG-3’
*IL29 (IFNL1)*	F: 5’-GGAGTTGCAGCTCTCCTGTC-3’ R: 5’-CAGCGGACTCCTTTTTGGGG-3’
*MX1*	F: 5’-CTTGTGAACGAAGATAAGTG-3’ R: 5’-TCTACCTCTGAAGCATCC-3’
*IFNL3*	F: 5’-TCGCTTCTGCTGAAGGACTGCA-3’ R: 5’-CCTCCAGAACCTTCAGCGTCAG-3’
*OAS1*	F: 5’-CAAGCTCAAGAGCCTCATCC-3’ R: 5’-TGGGCTGTGTTGAAATGTGT-3’
*TLR2*	F: 5’-TTGCAAGCAGGATCCAAAGGA-3’ R: 5’-CAAGACCCACACCATCCACA-3’
*IFITM1*	F: 5’-GGCTTCATAGCATTCGCCTACTC-3’ R:5’-AGATGTTCAGGCACTTGGCGGT-3’
*BST2*	F: 5’-CAGAGAAGGCCCAAGGACAA-3’ R:5’-GTCCGCGATTCTCACGCTTA-3’
*PPIA*	F: 5’-CGCGTCTCCTTTGAGCTGTT-3’ R: 5’-CCATAGATGGACTTGCCACCA-3’

F, forward; R, reverse.

### 2.3 Next generation RNA sequencing

Short-read cDNA libraries were synthesized using the TruSeq Stranded mRNA (Illumina) and subjected to paired-end sequencing (Illumina HiSeq 2000). The trimming software Trimgalore was used to preprocess fastq files. FastQC was used to generate QC statistics. The trimmed, paired-end reads were aligned using Salmon 1.3 against human genome hg38 and separately against RSV transcripts (32). Mapped paired-end reads for were counted in each sample using RNA-Seq by Expectation Maximization (RSEM). Contrasts were compared for virus infection and treatment conditions using DESeq2 (33).

### 2.4 Expression plasmids

The 3XFLAG-XBP1s cDNA was cloned in a lentiviral vector driven by a CMV promoter as previously described (28). The hIRF1 lentivirus expression vector in the pLV-tetO-CMV-SV40-Puro-LoxP expression plasmid was also previously described (34). Lentiviruses expressing FLAG-XBP1s (FXBP1s), the empty vector (pCT) and hIRF1 were generated by calcium phosphate precipitation transfection of HEK293T cells. The cells were cultured for 48 h and the virus-containing medium was collected, centrifuge-clarified and stored at -80 °C. Transduction ofhSAECs was performed in the presence of 10 μg/ml polybrene at an MOI of 2.0. The medium was changed, and cells cultured for an additional 24-48 h prior to further treatment.

### 2.5 Western immunoblot

Trypsinized cells were pelleted and washed with ice-cold phosphate-buffered saline (PBS). Cells were lysed in cell lysis buffer (10 mM Tris, pH 7.5, 100 mM NaCl, 1 mM EDTA, 1 mM EGTA, 20 mM Na_4_P_2_O_7_, 1 mM β-glycerol phosphate, 0.1% SDS, 0.5% sodium deoxycholate, 1% Triton X-100, 10% glycerol, 2 mM activated Na_3_VO_4_, 1 mM NaF and freshly added 1x protease inhibitor cocktail), and centrifuged at 16,000x g at 4 °C for 10 min. Proteins were then resolved on 4-15% Criterion TGX precast SDS-PAGE gels (Bio-Rad) and electro-transferred to PVDF membranes (Bio-Rad Trans-Blot Turbo transfer system). Primary antibodies (Abs) were anti-FLAG M2 (Sigma Aldrich F1804), anti-XBP1s (Clone 143F, BioLegend) and anti-TBP (TATA-box binding protein) Abs. TBP was used as a loading control. Blots were imaged and quantified using ImageJ.

### 2.6 Immunofluorescence microscopy

hSAECs were plated on coverslips, infected or treated as indicated, fixed with paraformaldehyde (4% vol/vol) and permeabilized with Triton X-100 (0.2% vol/vol). Afterwards, cells were blocked with 10% goat serum and incubated with primary anti-FLAG M2 Ab overnight at 4 °C. Afterwards, coverslips were washed and incubated with Alexa fluor-conjugated goat secondary Ab. After 1 h, cells were washed and mounted using ProLong Diamond Antifade Mountant with 4′,6-diamidino-2-phenylindole (DAPI, Thermo Fisher). The cells were visualized in an ECHO fluorescence microscope.

### 2.7 Cleavage Under Targets and Release Using Nuclease (CUT&RUN)

FLAG-XBP1s (FXBP1s) lentiviral-transduced cells (MOI = 2.0, 48 h) were prepared in triplicates. RSV infection was with MOI = 1.0, 24 h; pCT-transduced cells, mock infected were negative controls. Cells were trypsinized (4 x 10^6^ cells/point), and incubated in 1 ml of nuclear extraction buffer (20 mM HEPES, pH 7.9, 10 mM KCl, 0.1% Triton X-100, 20% glycerol, 1x cOmplete proteinase inhibitor, 1x protein phosphatase inhibitor cocktail and 0.5 mM spermidine) for 10 min on ice. Nuclei were pelleted at 600x g, 5 min at 4°C and washed in wash buffer (WB; 20 mM HEPES, pH7.5, 150 mM NaCl, 0.05% Triton X-100, 0.1% BSA, 1x cOmplete proteinase inhibitor, 1x protein phosphatase inhibitor cocktail and 0.5 mM spermidine). Prior to Ab binding, the isolated nuclei were nutated in WB containing 2 mM EDTA at 4°C for 5 min. The nuclei were resuspended in antibody buffer produced by diluting 5 μg anti-FLAG M2 Ab (Sigma) in 500 μl of detergent-free WB, and nutated overnight at 4°C. After washing 3X in 500 μl of WB on ice (10 minutes each time), the nuclei were incubated in 50 μl of WB containing 2.5 μl of EpiCypher pAG-MNase 20x stock (EpiCypher, NC) at 4°C for 1 h. After washing as above, chromatin cleavage was then conducted by incubating the nuclei in 150 μl of BSA-free WB containing 2 mM CaCl_2_ on ice for 1 h. The cleavage was terminated by adding 150 μl of stop buffer (300 mM NaCl, 20 mM EDTA, 4 mM EGTA and 0.5 ng of E. coli Spike-in DNA (EpiCypher, NC) per 150 μl). The samples were nutated for 1 h at 4°C, centrifuged at 16,000x g for 5 min at 4°C and the supernatant collected. DNA was extracted by phenol-chloroform, precipitated with glycogen and DNA fragments dissolved in 20 μl of 0.1x TE buffer.

DNA was quantitated by a Qubit fluorometer, CUT&RUN DNA libraries were prepared using NEBNext Ultra II DNA Library Prep Kit for Illumina (NEB, MA) per the manufacturer’s instruction, with modification in SPRI bead clearance of adaptor ligation and library amplification reactions to retain small-sized DNA fragments. The DNA library quality was confirmed by Agilent TapeStation HS DNA assay (Agilent, Santa Clara, CA, USA), and paired-end Illumina NGS was carried out on NovaSeq 6000 with 5 million reads per sample.

Quality metrics were generated using fastQC. Adapter trimming was by TrimGalore. Genome alignment to the GRCh38.p13 (hg38) genome assembly (NCBI) was by MACS2 (35). SEACR was used for peak calling with top 20% peaks (36).

### 2.8 Short hairpin RNA (shRNA) gene silencing

shRNA silencing was performed using lentivirus transduction for XBP1 or IRE1 (Sigma Mission shRNA lentiviral vector). Populations of transduced hSAECs were selected in 2 μg/ml puromycin. The most effective silencing lentivirus was identified by Q-RT-PCR and used in subsequent experiments. shRNA target sequences were: XBP1, 5’-GCCTGTCTGTACTTCATTCAA-3’; IRE1, 5’-GCAGGACATCTG GTATGTTAT-3’. Non-targeting luciferase shRNA lentiviral vector was used as negative control (Sigma, cat. SHC007).

### 2.9 IRF1 deletion

The lentivirus for IRF1 knockout was prepared with the lentiCRISPRv2 vector with the guide RNA (gRNA) sequence 5’- CACCTCCTCGATATCTGGCA-3’ targeting exon 4 of the human IRF1 gene (37). A549 cells were infected with virus supernatant in the presence of 10 ug/ml polybrene and selected 48 h later with 2 ug/ml puromycin. Empty lentiCRISPRv2 -transduced A549s were puromycin-selected and used as a negative control. IRF1 silencing was confirmed by Q-RT-PCR.

### 2.10 KRAB/dCAS9-mediated silencing of IRF1 Enhancer in hSAECs (CRISPRi)

The single guide (sg) RNAs were designed for IRF1 target regions using CHOPCHOP (http://chopchop.cbu.uib.no). A human genome non-targeting gRNA was used as a negative control. Synthesized oligonucleotides (Integrated DNA Technologies) were annealed and cloned into the Esp3I restriction sites of lentiviral expression vector Lenti_sgRNA_EFS_GFP (Addgene plasmid # 65656 (38)). Sanger sequencing confirmed sgRNAs insertion. The gRNA target sequences are non-targeting, 5’-GTTCCGCGTTACATAACTTA-3’, and IRF1 enhancer targeting, 5’- TCGGCGCGCAGGCACTCAGA-3’.

Lentivirus was produced in HEK293FT cells used to transduce target cells in the presence of 4 µg/mL polybrene (Sigma). For IRF1 Enhancer silencing, hSAECs were first transduced with the lentiviral vector pHR-SFFV-KRAB-dCas9-P2A-mCherry (Addgene plasmid # 60954 (38)). The vector expresses a fusion protein of mammalian codon-optimized Streptococcus pyogenes dCas9 (DNA 2.0) fused at the N terminus with the Kox1 KRAB domain and two SV40 nuclear localization sequences at the C terminus. mCherry-expressing hSAECs were transduced with the lentiviral sgRNA expression vector. Two days later, the dual mCherry and GFP-expressing cells were sorted and cultured for RSV infection experiments.

### 2.11 Two-step chromatin IP (XChIP)-Quantitative genomic PCR (Q-gPCR)

Two-step XChIP was performed using sequential protein crosslinking with DSG (2 mM, 45 min at 22°C) followed by protein–DNA cross-linking with formaldehyde (39). Equal amounts of sheared chromatin were immunoprecipitated (IPed). After overnight IP, complexes were collected using protein G-conjugated magnetic beads (40 μL, Dynal Inc), extensively washed and eluted in elution buffer for 15 min at 65 °C. Following de-crosslinking and phenol-chloroform DNA purification, gene enrichment was determined using region-specific PCR primers in Q-gPCR. The Q-gPCR primers specific for the Enhancer region of human IRF1: 5’-CTCCAAGTCATGTTCGGGGA-3’ (forward), 5’-AGCTAAGGGGTTTGAGGGTG-3’ (reverse). *SNAI1* primers were previously reported (19). The fold change of DNA in each IP was determined by normalizing to input DNA reference and calculating the fold change relative to unstimulated cells (40).

### 2.12 Statistical analyses

Statistical analyses of Q-RT-PCR assays were performed with Graph Pad Prism 9 (GraphPad Software, San Diego, CA). Results are expressed as mean ± SD. Normality and equal variance tests were performed to determine appropriate application of parametric statistical analyses. For multiple group experiments, ANOVA was used with *post-hoc* Tukey T-tests for group-wise comparison between treatments. P values < 0.05 were considered to be statistically significant.

Differential peak occupancy in CUT&RUN was determined using DIFFBIND v 4.2 (41) using DESEQ2 (33). Significance of differential occupancy was estimated by adjusted p values (pAdj) controlling for multiple hypothesis testing. Significance was met for pAdj <0.05. Enrichment analysis of known transcription factors was performed with HOMER software v 4.11 using position-weight matrices using hg38 as reference (42). Hierarchical clustering was using Z-score normalized transcripts per million (TPM) across rows using Euclidian distance.

## 3 Results

### 3.1 The UPR has complex effects on RSV transcription and innate signaling

Previous work has shown that IRE1α plays important roles in anti-viral defenses (43) as well as modifying the innate response to RSV replication (19). To understand this interrelationship in more detail, we explored effects of activation and inhibition of the IRE1α-XBP1s pathway on RSV transcription in human epithelial cells. For this analysis, we focused on telomerase-transformed *Scgb1a1* ^+^ human small airway epithelial cells (hSAECs), a cell type permissive for RSV infection and replication (19, 23). A body of evidence shows that hSAECs stably maintain genomic and proteomic innate signatures representative of primary SAECs in the basal and RSV-infected states (12), where RSV activates the UPR activating the hexosamine biosynthetic pathway, producing cell-state transition and extracellular matrix remodeling characteristic of *in vivo* RSV-infections (44–47).

**Figure 1:**
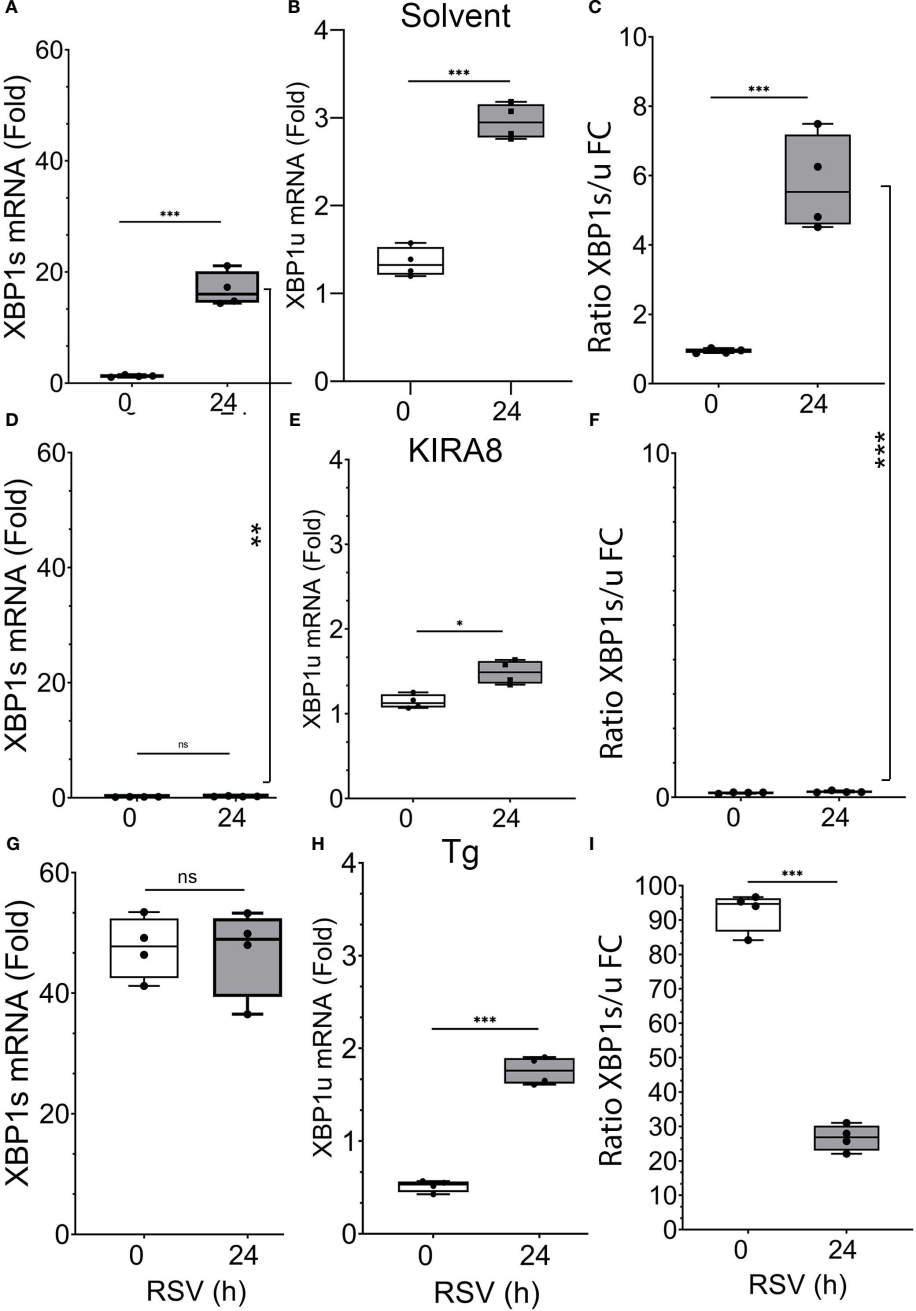
RSV activates XBP1s. hSAECs were pre-treated with solvent (DMSO) or selective IRE1α kinase inhibitor RNase attenuator (KIRA8, 10 μM) (31) for 2 h, followed by 3 h RSV adsorption in continued presence of DMSO or KIRA8 (MOI = 1.0). Separately, hSAECs were treated with solvent (DMSO) or 50 nM Tg to directly activate the UPR during the 3h RSV adsorption as above. After the 3 h RSV adsorption, cells were washed and either harvested (0 h) or cultured for additional 24 h in continued presence of solvent, KIRA8 or Tg. Q-RT-PCR was used to quantitate changes in XBP1s **(A, D, G)** and unspliced XBP1 (XBP1u) mRNAs **(B, E, H)**. The ratio of fold induction XBP1s to XBP1u was calculated **(C, F, I)**. Error bars are mean ± SD with four independent replicates. n.s., not signifiicant; *, P<0.05; **, P<0.01; ***, P<0.001; *post-hoc* analysis.

To understand the effects of IRE1α activation or inhibition on RSV replication and the innate response, hSAECs were pre-treated with vehicle (solvent) alone, or a selective IRE1α RNase inhibitor (IRE1αi, KIRA8 (31)) for 2 h to inhibit the UPR. Cells were then adsorbed with RSV for 3 h in the continued presence of vehicle or IRE1αi. In parallel, the UPR was activated by treatment with thapsigargin (Tg, 50 nM) administered during the RSV adsorption phase. Cells were washed and activation of the IRE1α-XBP1s pathway was analyzed by Q-RT-PCR by measurement of XBP1s formation as well as monitoring the expression of unspliced XBP1 (XBP1u) using our validated PCR assays (19). We observed that RSV replication induced a 16.9 ± 3-fold increase in XPB1s (P<0.001, *post-hoc*, (**Figure 1A**). A smaller, but significant induction of 2.9 ± 0.2-fold increase in XBP1u mRNA was also observed (**Figure 1B**), suggesting that XBP1 is regulated by both changes in gene expression as well as mRNA processing. This finding is consistent with our previous finding that XBP1 mRNA expression is autoregulated by XBP1s binding to its proximal promoter (19). However, the formation of XBP1s was relatively enhanced as shown by examining the ratio of the fold change inductions, where an increase in XPB1s over that of XBP1u is seen (**Figure 1C**). The potent effect of the IRE1αi is seen where both XPB1s, XBP1u mRNA are completely inhibited (**Figures 1D, E**), and there is no induction of XBP1s relative to XBP1u (**Figure 1F**). Finally, the 3 h Tg pre-treatment during the RSV adsorption was a potent inducer of *XBP1s* splicing, producing a 47 ± 7-fold increase in *XBP1s* over solvent-treated controls (**Figure 1G**), a 1.8 +/- 0.2-fold increase in XBP1u (**Figure 1H**), and 95-fold increase in XBP1s/u ratio (**Figure 1I**; this ratio is overestimated due to the reduction in XBP1u at 0 h). At 24 h the XBP1s/u ratio apparently falls, due to combined effects of XBP1u expression and cellular toxicity (**Figure 1I**). Nevertheless, these data indicated that RSV activated the UPR, but much less potently than Tg, and that the IRE1αi was highly effective antagonist of RSV-induced UPR activation.

**Figure 2:**
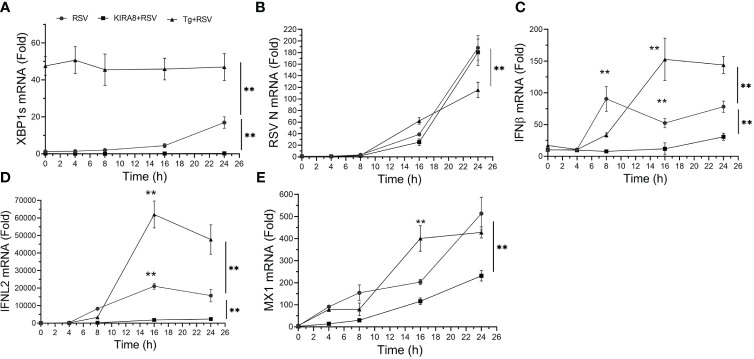
The IRE1α-XBP1s pathway has complex effects on RSV replication and the innate immune response. hSAECs, treated with solvent (DMSO) or selective IRE1α kinase inhibitor RNase attenuator (KIRA8, 10 μM) (31) for 2 h, were infected by RSV at an MOI of 1.0 for 3 h with continued presence of DMSO or KIRA8. Separately, hSAECs were infected by RSV (MOI=1.0) for 3 h in the presence of DMSO or 50 nM Tg activating the UPR. After 3 h viral adsorption, cells were washed and at 0 h or cultured for an additional 4, 8, 16 or 24 h in the presence of DMSO, KIRA8 or Tg as indicated. Q-RT-PCR was carried out for the mRNA levels of XBP1s **(A)**, RSV N **(B)**, IFNβ **(C)**, IFNL2 **(D)** and MX1 **(E)**. Error bars are mean ± SD with four independent replicates. **, P<0.01, *post-hoc* analysis.

To understand the dynamics of the innate response to RSV replication a time course of gene expression in cells with activated or inhibited IRE1α was conducted. As noted earlier, Tg was a potent inducer of XBP1s splicing throughout the 24 h time course (**Figure 2A**). In contrast, RSV induced a gradual increase in XPB1s formation, rising to 16.9 ± 3-fold at 24 h (P<0.001, *post-hoc*, **Figure 2A**). Expectedly, the IRE1αi reduced basal (uninfected) XBP1s to 15% that of untreated cells, and completely suppressed XBP1s formation throughout the time course (**Figure 2A**).

To understand the effect of the UPR on RSV transcription, the abundance of RSV N transcript was measured. Here a time-dependent increase in RSV transcription was observed where transcription was slightly increased in the Tg-treated cells after 16 h, but was significantly reduced at 24 h relative to the untreated and IRE1αi treated cells (188 ± 21-fold vs 115 ± 13 fold, P<0.01; **Figure 2B**). These data suggested that activation of the IRE1α pathway initially facilitated RSV transcription, but at later times, reduced its transcription. Finally, the IRE1αi-treated hSAECs had slightly less levels of RSV transcription relative to solvent treated cells at 16 h, but were indistinguishable at 24 h (n.s.; **Figure 2B**).

To examine how these changes affected the innate anti-viral pathway, we conducted kinetic measurements of type I and III IFNs and IFN stimulated genes (ISGs) over 24 h of RSV infection. We noted a complex effect of IRE1αi and Tg treatments on the ISG anti-viral response. In solvent-treated cells, the type I IFN, *IFNβ*, increased to 90 ± 19-fold at 8 h relative to mock-infected cells (P<0.001, **Figure 2C**), and was substantially inhibited by either IRE1αi or Tg (**Figure 2C**). By contrast, at 16 h and 24 h, the level of IFNβ expression in Tg-treated cells was significantly higher than that of solvent- or IRE1α treated cells. In particular, at 24 h, IFNβ expression in the Tg-treated cells was 144 ± 13-fold, whereas the solvent-treated cells, IFNβ expression was at 78 ± 9-fold. And by contrast, the level of IFNβ in the IRE1αi-treated cells was less than either treatment at all time points (**Figure 2C**).

We similarly explored the effect of UPR activation on RSV-induced type III IFN, *IFNL2*. In a manner similar to the effect on *IFNβ*, Tg treatment produced a delayed induction of *IFNL2*, peaking at 16 h at a level significantly higher than that of solvent-treated cells (47,700 ± 8,400 vs 15,700 ± 3,600-fold, P<0.01, **Figure 2D**). And, by contrast, IRE1αi significantly reduced *IFNL2* expression relative to solvent-treated hSAECs at all points in the time course (**Figure 2D**). Finally, the ISG, *MX1*, expression was enhanced by Tg at the 16 h time point, but not significantly different from solvent-treated cells at 24 h (**Figure 2E**). These data indicate to us that the Tg-induced UPR enhances type I and III IFN expression in response to RSV, resulting in reduced RSV transcription at 24 h of infection. And although IRE1αi reduces IFN expression, there is no detectable effect on RSV transcription at 24 h. Collectively these data illustrate a complex, dynamic relationship between the UPR, RSV replication and pathways of the innate response.

### 3.2 Identification of an IRE1α-XBP1s dependent innate regulatory network

To identify the direct effects of the IRE1α-XBP1 pathway on anti-viral response, we focused on the 24 h point, where RSV transcription is unaffected by IRE1αi, enabling us to control differences in viral transcription in the data analysis. Here, we integrated RNA-Seq profiling with chromatin occupancy analysis using CUT&RUN. Identification of XBP1s binding alone will not provide information on the functional regulation of target genes, and analysis of RNA expression alone is unable to differentiate direct targets vs indirect targets.

**Figure 3:**
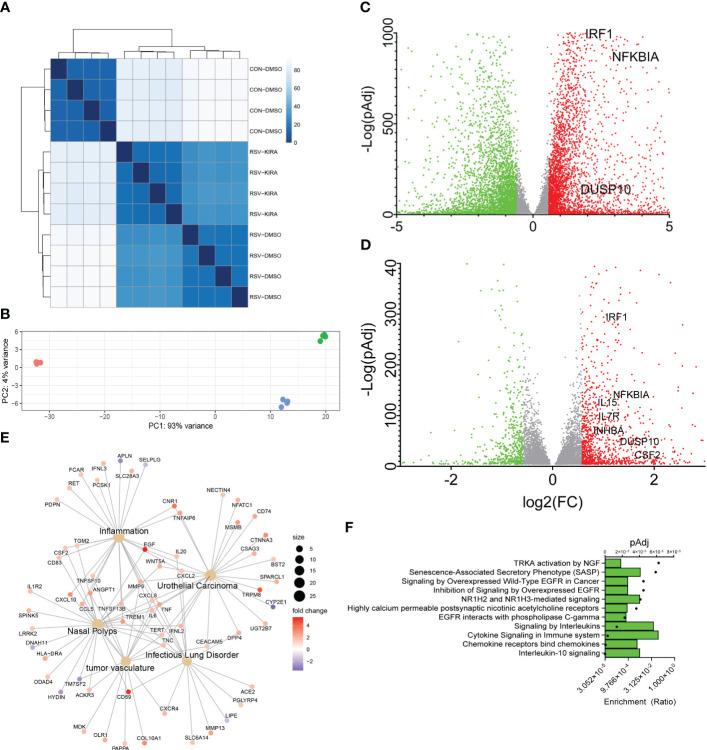
An IL10 cytokine network is mediated by IRE1-XBP1s signaling in RSV infection. **(A)** Correlation plots of individual RNA-seq experiments. Four independent replicates of short-read RNA-seq were analyzed from mock-treated, or RSV infected cells in the absence (DMSO) or presence of a selective IRE1α kinase inhibitor RNase attenuator (KIRA8, 10 μM) (31). Scale of correlation is shown in the inset. **(B)** Principal Component (PC) analysis of short-read RNA-seq replicates. Each point represents an independent replicate. Note the similar clustering of RSV and RSV_KIRA8 along PC 1, accounting for 91% of the experimental variance. **(C)** Volcano plot of differentially expressed genes (DEGs) in uninfected WT vs RSV infected cells. X axis, log_2_Fold Change of transcripts/million (TPM). Y axis, -log10 (adjusted p value using benjamini-hochberg, padj). Green symbols, genes downregulated by RSV infection; Red symbols, genes upregulated by RSV infection. Selected cytokine genes are labeled. **(D)** Volcano plot of RSV vs RSV_KIRA8 treated cells. X and Y axis are as in **(C)**. Green symbols, genes upregulated by KIRA8 in RSV infection; Red symbols, genes downregulated by KIRA8 in the setting of RSV infection. **(E)** Integrated network analysis of KIRA8-regulated transcripts in RSV infection. Cells are grouped by disease using Disease Ontology Semantic and Enrichment analysis (DOSE). Hub diameter (size) indicates the number of genes within the disease process. Node color indicates fold change in RNA expression by KIRA8 administration. **(F)** Gene Set Enrichment Analysis (GSEA) of KIRA8-regulated genes. Genes with 1.5-fold change in TPM and adjusted p-value of < 0.01 were analyzed. For each gene set, the fraction of genes represented in the pathway and the adjusted pValue (pAdj) for the top 10 overrepresented pathways are shown.

hSAECs were mock-infected or RSV infected for 24 h in the absence or presence of a IRE1αi using n = 4 independent biological replicates, and RNA-seq was conducted. After filtering and QC analysis, differential gene expression between groups was estimated using an established negative binomial distribution model (33). We found that the replicates were highly correlated with each other, falling into clearly separable RSV-infected, RSV + IRE1αi-treated and mock-infected (Control) treatment patterns (**Figures 3A, B**). Differentially expressed genes were those that demonstrated a 1.5-fold change and met an adjusted pValue (pAdj) cut-off of <0.05 to control for multiple hypothesis testing. Using this criteria, we compared gene expression in RSV vs mock-infected cells. Here, 1,538 genes were significantly downregulated and 1,723 genes upregulated by RSV infection (**Figure 3C**). Principal components analysis (PCA) showed RSV infection produced 93% of the variance of the data (**Figure 3B**), suggesting that RSV infection exerted the most profound effect on epithelial genome of the conditions tested. These findings are consistent with earlier findings demonstrating RSV produces a global effect on cellular gene expression programs (8, 9, 23).

To identify those genes regulated by IRE1α-XBP1s pathway, we next compared gene expression between the RSV and RSV+IRE1αi-treated groups. Interestingly to us, in analysis of the PCA plot, the effect of IRE1αi was detected only by the second principal component accounting for ~7% of the variance, indicating that the IRE1-XBP1s pathway accounted for a small fraction of the RSV-induced genomic response (**Figure 3B**). Application of the same 1.5-fold change and pAdj criteria, we found that 218 genes were inhibited by IRE1αi (**Figure 3D**). The IRE1αi-sensitive genes were subjected to semantic Disease Ontology analysis, and biological pathways by Panther Pathways. Prominent disease pathway nodes were identified as “nasal polyps”, “inflammation”, “tumor vasculature”, and “infectious lung disorder”, consistent with RSV infection. Importantly, a number of inflammatory and anti-viral cytokines were identified including *CXCL10, CD69, EGF, CXCL8, IFNL2, CSF2* and *TNF* (**Figure 3E**). Biological Pathway enrichment in Panther identified a predominance of “IL-10” and “cytokine signaling” pathways indicating that IRE1α-XBP1 signaling affected a broad network of cytokine networks in RSV infection (**Figure 3F**).

### 3.3 Identification of genes directly occupied by XBP1s binding

The RNA expression profiling includes genes directly regulated by XBP1s as well as those indirectly activated by RSV infection. To more precisely identify direct targets of XBP1s, we applied a validated CUT&RUN assay, where high resolution of XBP1s binding sites could be identified by high-throughput sequencing. Although CUT&RUN enables selective cleavage of XBP1s binding within a native chromatin environment, the antibody affinity is a critical component of success of this approach. Available XBP1s antibodies have either poor affinity or inherent cross-reactivity with XBP1u and/or unrelated proteins, which makes the interpretation of binding sites using native antibody enrichment problematic.

**Figure 4:**
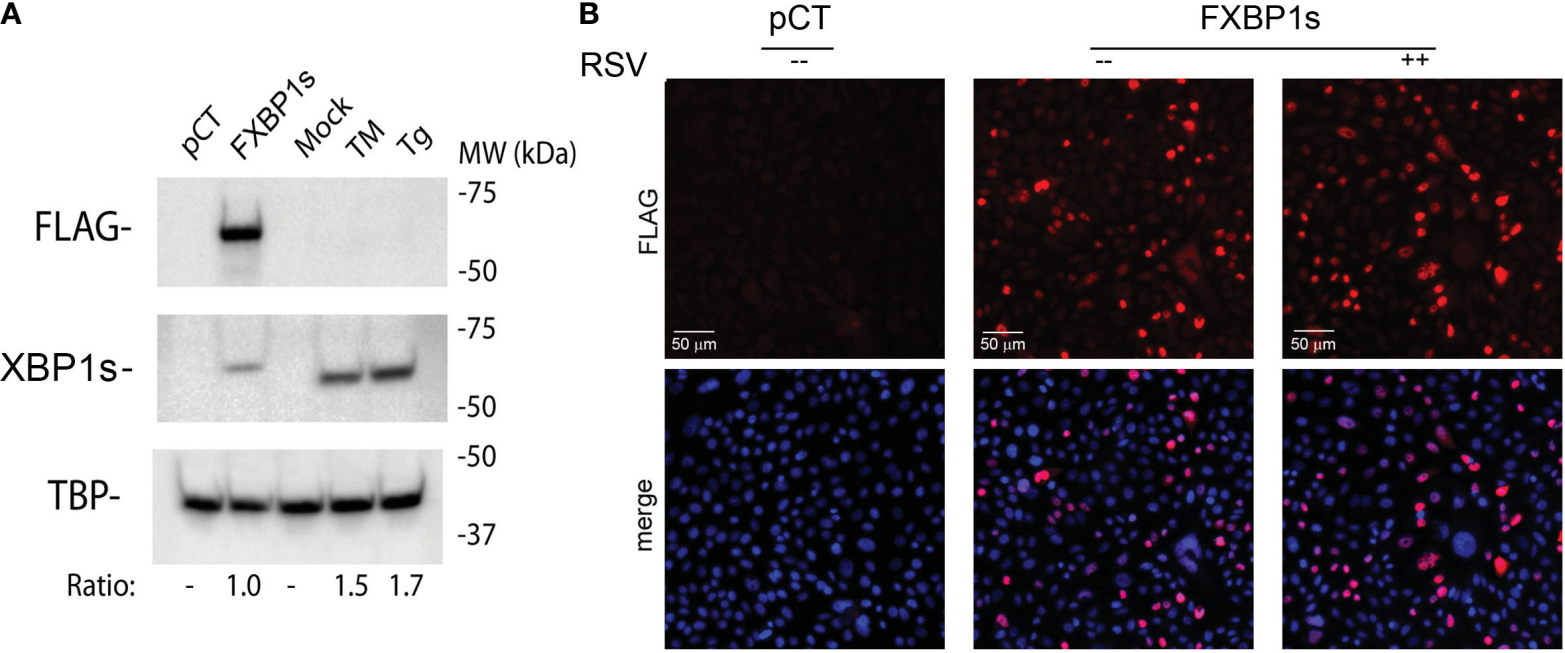
Expression of functionally active FLAG epitope-tagged XBP1s (FXBP1s). **(A)** FXBP1s expression. Western immunoblot of hSAECs transduced with empty (pCT) or FXBP1s-expressing lentiviral vector at an MOI of 2.0 for 48 h, or mock-treated or treated with tunicamycin (TM, 0.5 μg/ml for 8 h) or thapsigargin (Tg, 50 nM for 6 h) as indicated. Nuclear extracts were prepared and stained with anti-FLAG M2 Ab (top) or anti-XBP1s antibodies (middle panel). TATA box binding protein (TBP) was used as loading control (bottom panel). Molecular weight (MW) markers (in kDa) are shown. Ratio, the relative intensities of XBP1s immunoblotting signals normalized to TBP. Note the selective anti-FLAG M2 staining of ~60 kDa protein encoded by the FXBP1s expression vector and the significantly higher reactivity of the anti-FLAG M2 antibody (Sigma) than the anti-XBP1s antibody (BioLegend). Note TM and Tg induce significantly higher levels of XBP1s than that produced by FXBP1s transduction. **(B)** Immunofluorescence microscopy was performed to assess FXBP1s transduction efficiency and nuclear translation. Cells were infected with empty (pCT) or FXBP1s-expressing lentivirus (MOI=2). 24 h later, cells were mock or RSV infected (MOI=1) for an additional 24 h before fixation and staining with anti-FLAG M2 (red). Nuclei were counter-stained with DAPI (blue). Top panel, anti-FLAG M2 staining; Bottom panel, anti-FLAG M2 and DAPI staining are merged. Scale bar of 50 μm is shown. Note the >50% transduction of the cell population and nuclear localization.

To address this problem, we validated expression of a 3X FLAG epitope-tagged XBP1s (FXBP1s), where FXBP1s expression levels can be controlled by the multiplicity of infection (MOI). We first confirmed FXBP1s expression levels were comparable to that produced by activation of the endogenous UPR. Here, FXBP1s expression levels were compared to endogenous XBP1s produced by tunicamycin (TM) or thapsigargin (Tg) treatment. FXBP1s was quantified using anti-FLAG, anti-XBP1s Abs and anti-TATA Box Binding Protein (TBP) Ab staining in Western blot (anti-TBP staining was loading control). Relative to empty virus (pCT)-transduced cells, FXBP1s was detected by anti-FLAG Ab as a ~60 kDa nuclear protein. Importantly, this protein cross-reacted with anti-XBP1s Ab (**Figure 4A**, middle panel). Staining was undetectable in untreated or pCT-transduced cells (**Figure 4A**, top). We confirmed that TM or Tg treatment similarly induced robust accumulation of nuclear XBP1s, and noted that the lentivirus-encoded FXBP1s expression was ~60% of the XBP1s levels induced by TM (**Figure 4A**, middle panel). In parallel, immunofluorescence staining and microscopy confirm that the FXBP1s protein was concentrated in the nucleus, determined by co-localization with the nuclear DAPI marker, and identifiable in >50% of the cells (**Figure 4B**). These data indicate that FXBP1s expression was physiologically comparable to that produced by endogenous UPR activation by the IRE1α RNase and localized to the nucleus.

### 3.4 Profiles of XBP1s binding to the hSAEC genome

**Figure 5:**
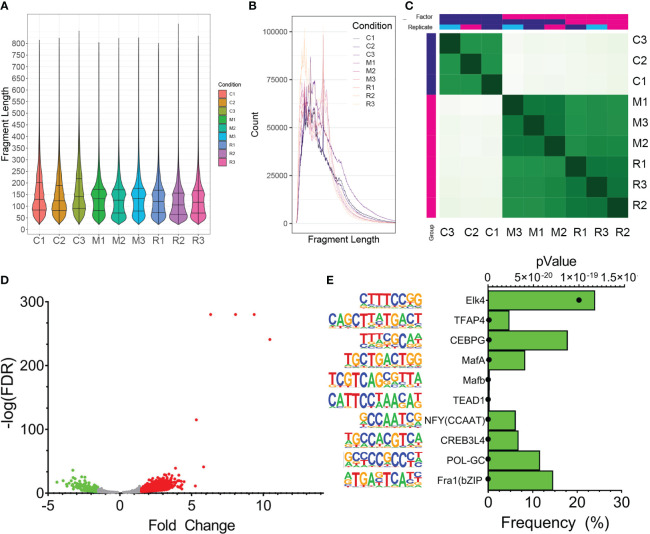
CUT&RUN Analysis of FLAG-XBP1s Binding. **(A)** CUT&RUN analysis was applied to pCT (C1-C3) or FXBP1-transduced cells in the absence (M1-M3) or presence of RSV infection (R1-R3; MOI =1, 24h). Shown are violin plots of fragment length distribution of cleavage fragments after removal of adapters. Note the distinct 70 and 180 nt pattern in the M-series and R-series for FXBP1s fragments that are absent in the nonspecific cleavage pattern of the control series. **(B)** Linear plot of fragment length of CUT&RUN data illustrating the 180 nt cut pattern, consistent with nucleosomal distribution. **(C)** Correlation plots of individual CUT&RUN binding. Each individual replicate from empty vector pCT (CON1-CON3) or FXBP1 transduced cells in the absence (MM1-MM3) or presence of RSV infection (RM1-RM3; MOI =1, 24h). Note the high cross-correlation of the MM1-MM3 and RM1-RM3 with each other in the same replicate treatment that are distinct from that of control. **(D)** Volcano plot of FXBP1s peaks in uninfected vs RSV infected hSAECs. Differential analysis of FXBP1s binding was determined after normalization to library depth and differential analysis using DIFFBIND. X axis, Fold Change of binding occupancy. Y axis, -log10(adjusted p value using benjamini-hochberg, padj). Green symbols, FXBP1s peaks reduced by RSV infection; Red symbols, FXBP1s peaks upregulated by RSV infection. **(E)** Motif enrichment of FXBP1s binding sites. Motifs are rank ordered by the frequency of binding sites. On the left is the sequence logo followed by the JASPAR matrix name. For each, the p value of enrichment is also shown (symbol). Note enrichment of Fos-related protein 1 (Fra1) and GC sequences (POL-GC, CREB and NFY).

To identify genomic binding sites of XBP1s and determine whether RSV influences its genomic binding patterns, CUT&RUN profiling was performed in empty vector-transduced cells, FXBP1s transduced cells mock-infected with RSV and FXBP1s transduced cells infected with RSV. The NGS sequencing reads were subjected to quality control, which indicated high-confidence base calling of >150 base pairs with Phred scores > 30 (not shown). After trimming sequencing primers, numbers of counts, library size, fraction of reads in peaks (FRiP) and fragment size were examined. The data were mapped to 1,226,706 intervals, with FRiP of 0.28 ± 0.022 (**Table 2**). We observed that control fragment sizes were monotonically distributed at ~90 nt in both violin plots (**Figure 5A**), and by histogram (**Figure 5B**). By contrast, both the mock infected and RSV infected FXBP1s-transduced cells produced fragment sizes binomially distributed at ~70 and ~160 nt in length (**Figures 5A, B**). We interpreted this analysis to indicate our data showed consistent, high-quality reads across replicates with anticipated inter-nucleosomal cleavage pattern.

**Table 2:** Fractions of reads in peaks (FRiP).

ID	Antibody	Condition	FXBP1s	Replicate	Intervals	Reads	FRiP
*C1*	FLAG	Mock	pCT	1	1226706	7768023	0.24
***C2***	FLAG	Mock	pCT	2	1226706	7742521	0.24
***C3***	FLAG	Mock	pCT	3	1226706	10792713	0.27
***MM1***	FLAG	Mock	FXBP1s	1	1226706	9299732	0.27
***MM2***	FLAG	Mock	FXBP1s	2	1226706	8792937	0.3
***MM3***	FLAG	Mock	FXBP1s	3	1226706	959858	0.3
***RM1***	FLAG	RSV	FXBP1s	1	1226706	7914061	0.29
***RM2***	FLAG	RSV	FXBP1s	2	1226706	9080509	0.29
***RM3***	FLAG	RSV	FXBP1s	3	1226706	11009478	0.29

For each CUT&RUN sample, the FRiP was calculated and tabulated below. pCT, empty lentivirus, FXBP1s, FLAG-XBP1s expressing lentivirus.

Correlation analysis was used to establish replicate similarity. Here we found that the DNA fragments co-clustered by treatment type, with empty vector-transduced, mock-infected controls (C1-C3) clustering together and distinctly apart from either FXBP1s-transduced, mock-infected (MM1-MM3) or FXBP1s-transduced, RSV-infected replicates (RM1-RM3, **Figure 5C**). Statistically significant peaks occupied by FXBP1s were identified using DESEQ2 in DiffBind where FXBP1s bound 4,252 peaks in mock infected-FXBP1s expressing cells vs controls (the DESEQ2 pAdj <0.05 was used as a cut-off to accommodate for multiple hypothesis-testing). The occupancy and statistical confidence for the 4,252 peaks different between MM1-MM3 vs. C1-C3 were analyzed by Volcano plot where the number of increased peaks outnumbered those decreased, as well as the magnitude of fold change was skewed, with a greater number of high confidence peaks appearing in the MM samples vs. nonspecific peaks in the controls (**Figure 5D**).

Previous work has found that XBP1s binds to pleiotropic sequences, such as the unfolded protein response element (UPRE) and ER stress response element (ERSE) motifs in a manner determined, in part, by heterotypic interactions with other bZIP DNA-binding proteins that are under cell-type, differentiation-state and stimulus-dependent control (29, 30, 48). To first infer the binding sites enriched in FXBP1s-bound fragments in mock-infected epithelial cells, we conducted motif enrichment analysis on the 4,252 high-confidence FXBP1s peaks by scanning for 441 known transcription factor binding by determining binding probability using position weight matrices (42). We observed enrichment of >20 sequence motifs relative to background sequences. The top 10 ranking peaks identified included Fos-related antigen (Fra 1) motifs (containing the TGA-G/C-CTCA core recognized by Jun factors) present in 15% of all sequences, GC-box (containing GCCCCGCCC sequences found in 11% of sequences) and CREB motifs (GCCACGT core found in 6% of sequences; **Figure 5E**). These data suggest that FXBP1s interacts with DNA binding domains enriched in AP-1/CREB motifs in the absence of RSV infection.

**Figure 6:**
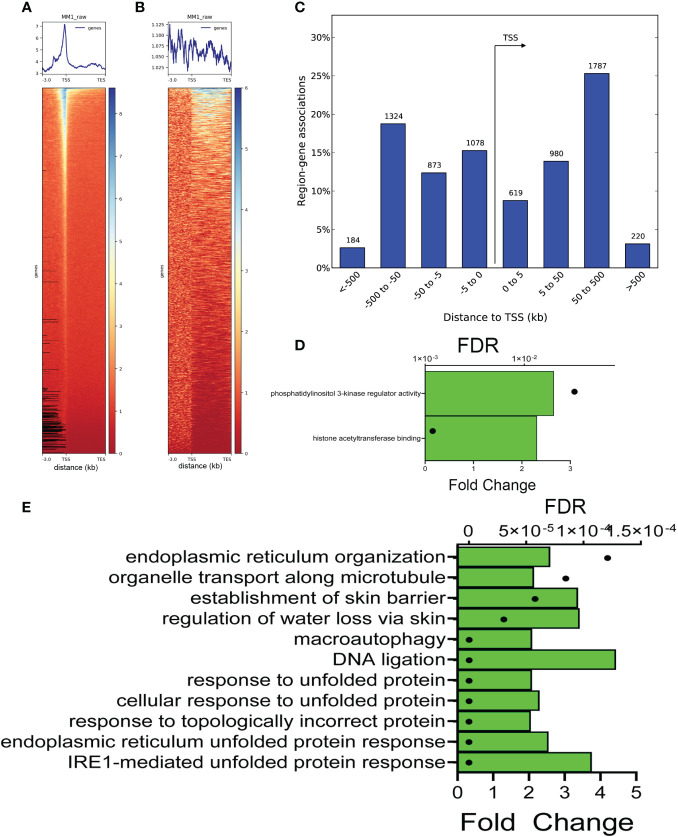
Annotation of FXBP1s binding sites. **(A)** XBP1s binding is enriched on proximal promoters. Clustered heatmap of XBP1s peaks in mock-RSV infected cells aligned with transcription start sites (TSS) of annotated genes normalized for gene length. Note the peak enrichment is ± 500 bp upstream and downstream of the gene TSS, and sharply falls outside this window. **(B)** Clustered heatmap of XBP1s peaks within introns and gene bodies. Note the lack of concordance over other gene regions. **(C)** Histogram of XBP1s peaks relative to TSS binned by distance to TSS in kilobasepairs (bp). Note the sharp drop-off in peak numbers >500 kbp upstream of TSS and >500 bp downstream of TSS. **(D)** GO Molecular Function of genes with FXBP1s-bound regulatory elements in mock-infected cells. GREAT analysis of genome ontology molecular function. Two major molecular functions were identified: phosphatidylinositol 3-kinase (PI3K) regulator activity, and histone acetyltransferase binding. For each Molecular Function, the binomial false discovery rate (FDR) is shown (symbol) and binomial enrichment (Enrichment) is plotted (green bar). **(E)** GO Biological Pathway of genes with FXBP1s-bound regulatory elements in mock-infected cells. Top 10 GO Biological Pathways for FXBP1s binding sites are shown with FDR and binomial enrichment as above. Note most significant pathways are in IRE1-mediated response, and ER/unfolded protein response and lack of IL10 cytokine enrichment expected by the RNA-seq functional analysis.

To identify the preferential location of FXBP1s binding sites in mock-infected cells, a heat map was constructed relative to length-normalized genes. A strong peak in the proximal promoters was identified (**Figure 6A**); by contrast, a significantly weaker association of binding over gene bodies was seen between the transcriptional start sites (TSS) and transcriptional end sites (TES) (**Figure 6B**). To identify biological processes, we next analyzed the FXBP1s-binding peaks using Genomic Regions Enrichment of Annotations Tool (GREAT) (49). GREAT maps CUT&RUN peaks to regulated genes by incorporating chromatin interaction and gene proximity information into a rule-based algorithm, improving the detection of biologically relevant peaks (49). In a manner consistent with our heat map analysis, GREAT mapped the majority of FXBP1s peaks to ± 500 kbp centered on the TSS of regulated genes (**Figure 6C**). Collectively these data indicate that FXBP1s primarily binds 5’ regulatory elements in hSAECs.

Two major Genome Ontology (GO) biological processes were identified by GREAT, that included histone acetyl transferase binding and PI3K regulatory activity (**Figure 6D**). Peaks were also analyzed by GO molecular pathways. Strikingly, 6 of the top 10 GO molecular pathways were highly enriched in “IRE1-mediated UPR”, “ER UPR”, and “UPR” (**Figure 6E**). In addition, we noted that these pathways lacked IL10 cytokine signaling that represented the dominant functions identified by RNA Seq (cf **Figures 6E**, **2F**).

### 3.5 Characteristics of RSV-modulated XBP1s binding motifs

**Figure 7:**
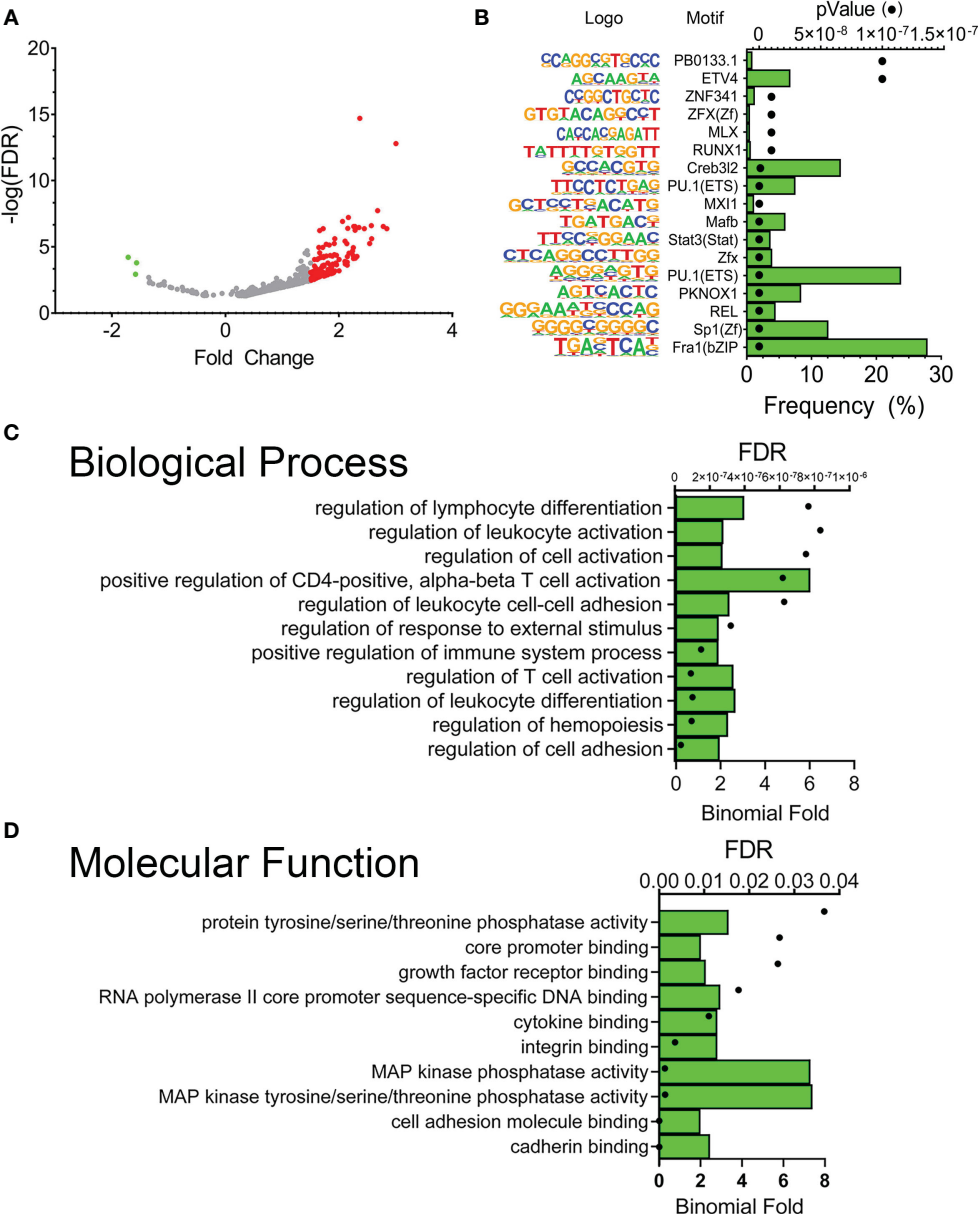
RSV modulates a subset of FXBP1s binding elements. **(A)** Volcano plot of FXBP1s peak abundance in RSV infected FXBP1s-expressing cells vs mock-infected FXBP1s-expressing cells. Note the smaller number of sites that change occupancy (250), with the majority increasing occupancy after RSV infection (red). **(B)** Motif enrichment of FXBP1s genomic sequences. Shown are the top 10 enriched DNA binding motifs in the DNA sequences bound by FXBP1s. On the left is the sequence logo, matrix name. Green bars show the frequency of the motif in the entire data set, and the symbols show the enrichment p value. Note enrichment of innate driven transcription factors, Fra1/activator protein 1 (AP1) and RelA. **(C)** GO Biological Pathway of genes with FXBP1s-bound regulatory elements in RSV-infected vs mock-infected cells. Top 10 GO Biological Pathways for FXBP1s binding sites identified by GREAT are shown with FDR and binomial enrichment as above. **(D)** GO Molecular Function of XBP1s-bound regulatory elements in RSV-infected vs mock-infected cells. Note enrichment of cadherin binding and hemopoiesis/leukocyte differentiation.

Our experimental design also enabled us to compare differences in XBP1s occupancy between RSV-infected vs. mock-infected FXBP1s cells. Only 786 peaks were different between these two groups, which were also asymmetrically skewed towards increased binding after RSV infection (**Figure 7A**). *De novo* motif analysis of these RSV-modulated peaks showed that these sequences were enriched in Fra-1 motifs (now present in 28% of total peaks) and appearance of REL targets (5% of total peaks), along with SP1-box sequences (containing the CCGCCC core; (**Figure 7B**). These data suggest enrichment of XBP1s binding to AP1 and RELA sequences activated by RSV infection.

The enrichment of GO pathways for the RSV-modulated XBP1s sequences was also analyzed by GREAT. Here we observed that RSV-modulated peaks regulated gene networks controlling “regulation of cell adhesion”, “regulation of hemopoiesis”, “regulation of leukocyte differentiation” and “T-cell differentiation” (**Figure 7C**). The most enriched GO molecular functions included “cadherin binding” and “MAP kinase tyrosine/serine/threonine phosphatase activity” (**Figure 7D**). These data indicated that RSV modulated XBP1s binding to a small subset of genes controlling cell adhesion and cytokine activity.

### 3.6 A core of directly XBP1s-regulated genes includes the anti-viral IRF1 transcription factor

We identified individual genes contained within the GO pathways, and found a common core set of cytokine regulatory genes that were directly regulated by XBP1s. These genes include interferon regulatory factor (*IRF*)*1, CSF2, NFKB1A, DUSP10*, and *IL15*. IRF1 is a ubiquitous and inducibly expressed nuclear transcription factor that maintains the basal transcription of a suite of antiviral ISGs (50). NFKBIA is a cytoplasmic inhibitor of IKK-RELA pathway controlling RELA translocation, mediating inflammatory cytokine activation in RSV-infected cells (51). DUSP10 is a MAP Kinase phosphatase that inactivates p38 and JNK implicated in RSV disease (52). Because this group of cytokine regulators have broad impact on innate signaling, we focused on this core set of genes, examining their expression patterns and sites of XBP1s binding.

**Figure 8:**
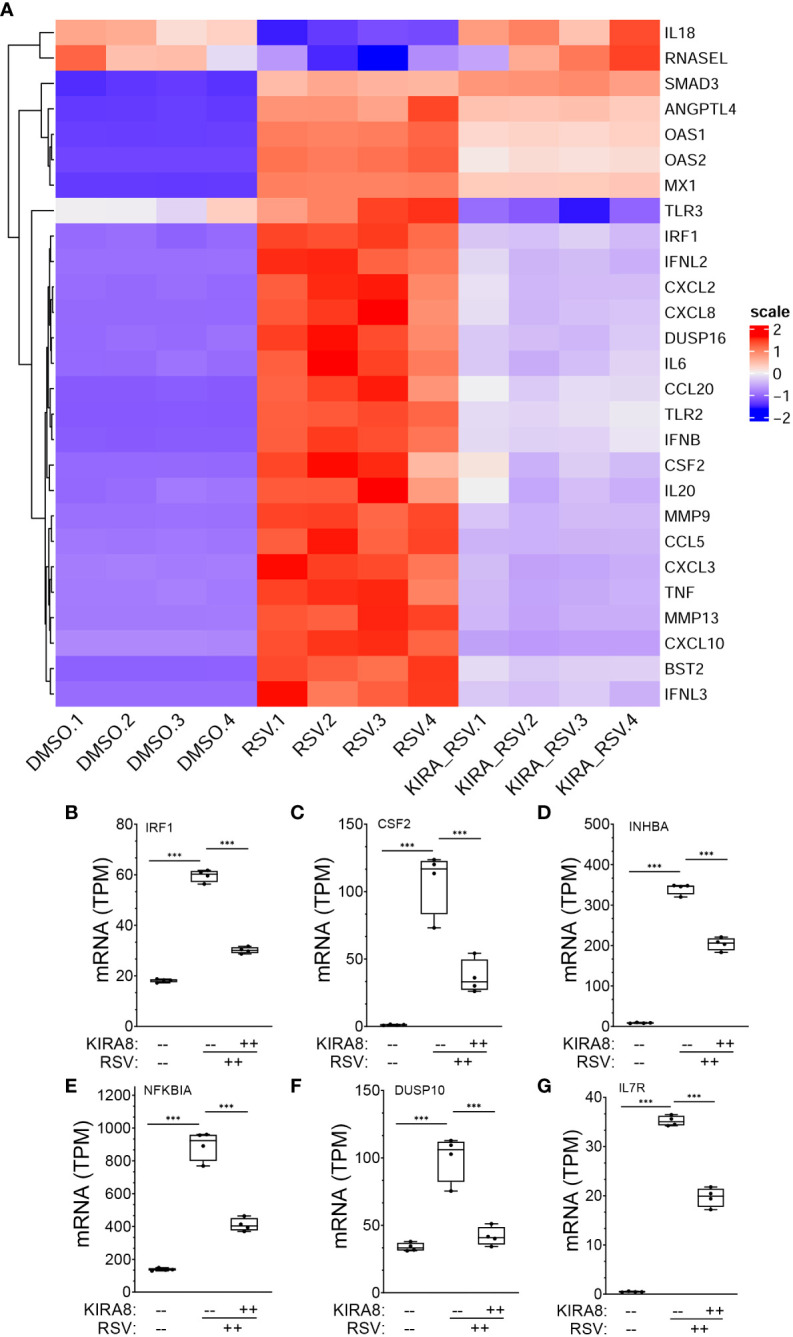
mRNA expression changes in XBP1s-bound cytokine regulators. **(A)** Hierarchical cluster of IL10 signaling and cytokine genes in RSV-infected hSAECs (from the GSEA classification in **Figure 1F**). Genes are organized by row, treatment conditions by column. Color indicates row-wise Z-score. Dendrogram is Euclidian distance. Scale indicates z-score statistic for each condition. Note the major cluster are type I and III IFN and IFN stimulated genes (ISGs) whose expression are increased by RSV infection. **(B-G)** Expression changes for genes directly regulated by XBP1s. For each gene shown, plotted are transcripts per million (TPM) from mock infected, RSV infected or RSV + IRE1α inhibitor (KIRA8) treated cells. Genes plotted are: **(B)** Interferon Regulatory Factor 1 (IRF1); **(C)** Colony Stimulating Factor 2 (CSF2), **(D)** Inhibin Subunit Beta A (INHBA); **(E)** NFKB Inhibitor Alpha (NFKBIA); **(F)** Dual Specificity Phosphatase 10 (DUSP10); and **(G)** Interleukin 15 (IL15). Box plots are 10-90% interquartile range with mean indicated by horizontal line. Each symbol is an independent replicate. Error bars are ± SD. ***, P<0.001, *post-hoc* analysis.

To further understand the effects of dysregulation of the IRE1α-XBP1s pathway on direct *vs* indirectly regulated genes, we examined cytokine expression profile of 27 genes identified within the IL-10/cytokine signaling pathway using hierarchical clustering. The expression values of these genes were normalized by Z-score and plotted (**Figure 8A**). Here, the majority of IFNs (*IFNB, IFNL2*) and IFN-stimulated genes (ISGs), such as 2’-5’-Oligoadenylate Synthetase 1 (*OAS*)-1/2, MX Dynamin Like GTPase 1 (*MX1*), toll like receptor (*TLR*)*3, CXCL2* co-clustered with *IRF1* as RSV-upregulated genes. We noted that expression of this cluster was significantly reduced by the IRE1αi treatment. By contrast, the expression of only 2 genes, *IL18* and *RNASEL* were reduced by RSV infection, and restored to baseline after inhibition of IRE1α (**Figure 8A**). Collectively these data indicate that the robust induction of ISG gene pathway is dependent on IRE1α-XBP1s signaling in RSV infection.

Expression patterns of the directly activated cytokine regulatory factors were analyzed in more detail. In mock-infected hSAECs, *IRF1* mRNA was expressed 18.2 ± 0.7 transcripts per million (TPM), increasing by 3.2-fold to 59.7 ± 2.3 TPM after RSV infection (P <0.0001, *post-hoc* t test. **Figure 8B**). Treatment with IRE1αi reduced *IRF1* mRNA to 30.2 ± 1.2 TPM (P <0.0001. **Figure 8A**). Similarly, *CSF2* mRNA increased from 1.2 ± 0.3 TPM to 108 ± 23.4 TPM after RSV, and was reduced to 36.6 ± 12.4 TPM after IRE1αi treatment (P <0.0001, both contrasts, *post-hoc* t-test, **Figure 8C**). Similarly, RSV induced *INHBA* expression by 40-fold, *NFKBIA* by 6.5-fold, *DUSP10* by 3-fold, and *IL15* by 5.3-fold, all of which were significantly inhibited by IRE1αi (**Figures 8D–G**).

### 3.7 FXBP1s directly binds to the core network of cytokine regulatory factors

To better understand how XBP1s transactivates the cytokine regulatory factors, we mapped XBP1s-associated fragmentation patterns from the CUT&RUN analysis to the *IRF1, CSF2, INHBA, NFKBIA, IL15*, and *DUSP10* genomic loci; these are displayed using the IGV viewer. In this visualization, we also displayed Lys-acetylated H3K27 (H3K27Ac) peaks H3K27Ac marks as a measure of transcriptionally active chromatin (53).

**Figure 9:**
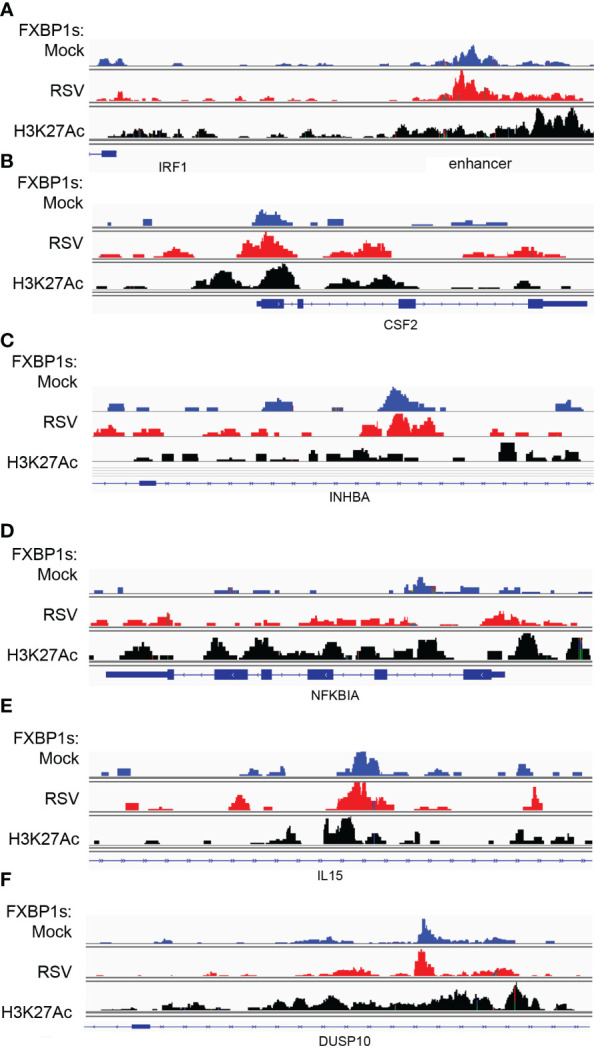
RSV modulates FXBP1s binding on cytokine regulatory core. Integrated genomics viewer (IGV) of individual XBP1s peaks. Top track, mock-infected cells (blue); middle track, RSV infected (red) cells; Bottom track, H3K27Ac peaks corresponding to open/activated chromatin. Blue boxes at bottom correspond to gene exons; arrows indicate direction of transcription. **(A)** *IRF1* 5’ region. Note major peaks are interacting with the 5’ upstream IRF1 enhancer and, to a lesser extent, the proximal promoter around the TSS. **(B)** *CSF2* gene. FXBP1s peaks are distributed on promoter, Exon 2/Intron 2 and 3’ regulatory region. **(C)** *INHBA*. **(D)** *NFKBIA*. **(E)** *IL15*. **(F)** *DUSP10*.

In the absence of RSV infection, XBP1s binds primarily to a 5’ regulatory element of the *IRF1* gene, with few peaks located in the proximal promoter (**Figure 9A**, top track). By contrast, RSV enhanced binding on the enhancer changing the envelope of distribution of cleavage sites with broadly increased loading on the enhancer and further 5’ upstream (Padj<0.05, DESEQ2, **Figure 9A**, middle track). We also noted that this region lies within open chromatin with broadly distributed H3K27Ac peaks (**Figure 9A** bottom track).

Similar patterns of constitutive and increased loading were observed for the proximal promoter of *CSF2* (**Figure 9B**), the intron of *INHBA* (**Figure 9C**), the proximal promoter and gene body of *NFKBIA* (**Figure 9D**), the intron of *IL15* (**Figure 9E**) and the *DUSP10* intron (**Figure 9F**). We conclude that FXBP1s binds to the proximal promoters of this group of cytokine regulators within transcriptionally active chromatin domains.

### 3.8 IRF1 mediates type III IFN expression and a subset of ISGs in RSV infection

The discrepancy between the RNA-seq and small number of core regulators identified as direct XBP1s targets suggested to us that the core regulators may be involved in the regulation of downstream gene regulatory networks.

**Figure 10:**
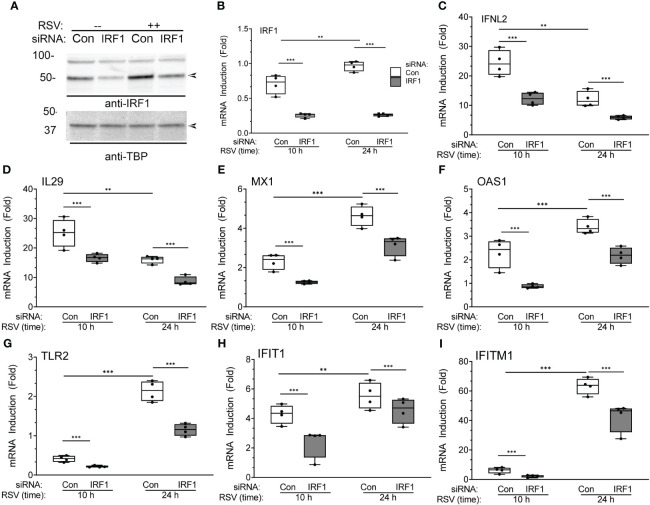
IRF1 is a regulator of XBP1s-dependent IFN gene regulatory network: effects of IRF1 silencing. **(A)** Western immunoblot of IRF1 in hSAECs transfected with scrambled (Scr) or IRF1-targeting siRNA. Cells were extracted after mock **(-)** or RSV infection (MOI=1, 24 h). Top panel, IRF1 staining. Specific IRF1 staining is seen at 50 kDa (arrow). Bottom, TATA-binding protein (TBP) staining is internal control. Left, molecular weight markers (in kDa). **(B-I)** Q-RT-PCR analysis of hSAECs after IRF1 silencing. hSAECs were siRNA transfected and RSV infected for 10 or 24 h and gene expression determined by Q-RT-PCR. Shown are 10-90% interquartile ranges of mRNA fold change relative to mock infected hSAECs. Gene expression is plotted for: **(B)** *IRF1* mRNA; **(C)** *IFNL2* mRNA; **(D)** *IL29* mRNA; **(E)** *MX1* mRNA; **(F)** *OAS1* mRNA; **(G)** *TLR2* mRNA; **(H)** *IFIT1* mRNA; **(I)** *IFITM1* mRNA. **, P<0.01, ***, P<0.001, *post-hoc* analysis.

To explore this phenomenon, we focused on the role of IRF1 in RSV induced type I-III IFN expression. For this purpose, we depleted IRF1 selectively using IRF1 knockdown. hSAECs transfected with scrambled or IRF1-selective siRNA were mock- or RSV infected (MOI=1, 24 h) and the abundance of IRF1 was determined by Western immunoblot. A specific 50 kDa IRF1 band was detected in mock infected cells that increased 2-fold in response to RSV infection (**Figure 10A**). The abundance of IRF1 was depleted by RNAi by >50% in mock infected cells, and substantially reduced in RSV infected cells to control levels. Confirmation of knockdown was confirmed by measurement of *IRF1* mRNA by Q-RT-PCR (**Figure 10B**). The same samples were assayed for type I and III IFNs and ISGs. We observed that *IFNL2* sharply increased to a 25-fold increase 10 h after infection and fell to 12-fold at 24.h. This induction was significantly reduced by IRF1 knockdown at both 10 and 24 h (**Figure 10C**). We observed a similar rapid *IL29* mRNA induction with significant inhibition by IRF1 silencing. The expression of ISGs, *MX1, OAS1, TLR2, IFIT1 and IFITM1* demonstrated peak expression at 24 h, and were substantially reduced by IRF1 depletion (**Figures 10D–I**, respectively). Collectively, these data indicate that IRF1, a direct XBP1s target, is an upstream mediator of type I-III IFN-ISG pathway.

**Figure 11:**
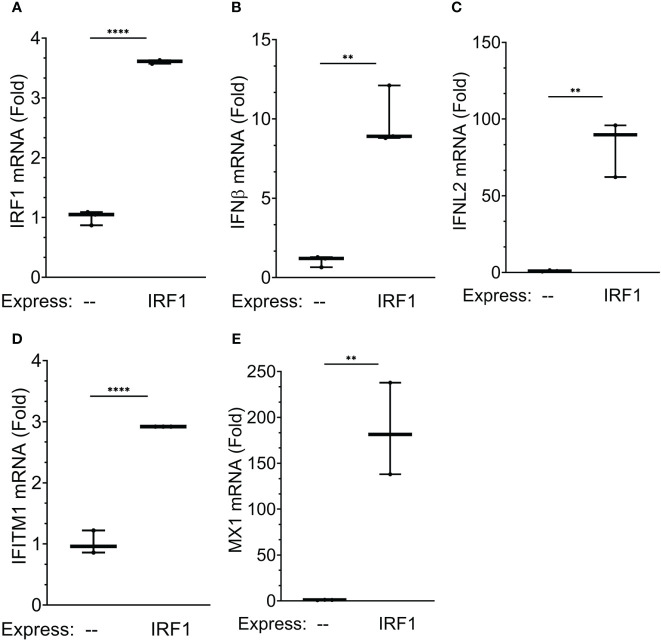
IRF1 is a regulator of XBP1s-dependent IFN gene regulatory network: effects of IRF1 expression. To examine the effects of physiological IRF1 expression, IRF1 expression was conducted in IRF1^-/-^ A549 cells generated by CRISPR/Cas9 site-directed recombination. Shown are 10-90% interquartile ranges of mRNA fold change relative to mock infected hSAECs. Gene expression is plotted for: **(A)** *IRF1* mRNA; **(B)** *IFNB* mRNA; **(C)** *IFNL2* mRNA; **(D)** *IFITM1* mRNA; and **(E)** *MX1* mRNA; **, P<0.01, ****, P<0.0001, *post-hoc* analysis.

To address the direct effect of IRF1 on ISG expression, we explored the effect of IRF1 expression. Recognizing that earlier studies found that high levels of ectopic IRF1 induces programmed cell death (54), we developed a physiological IRF1 expression system. Here, hIRF1 was expressed in IRF1-deficient A549 cells produced by CRISPR/Cas9-directed recombination (A549 cells are a well-established model for study of RSV-induced innate responses and were used because these cells tolerated multiple passages required for selection after CRISPR/Cas9-directed recombination). IRF1^-/-^ cells were transduced with empty lentivirus or hIRF1-expressing lentivirus expression of type I and III IFNs quantitated. Transduction with the hIRF1 vector produced a 3.6 ± 0.04-fold increase in *IRF1* expression, consistent with a physiological induction and produced no discernable programmed cell death (**Figure 11A**). Importantly, IRF1 expression induced a 9.9 ± 0.04-fold increase of *IFNβ* mRNA (**Figure 11B**); a 83 ± 18-fold induction of *IFNL2* mRNA (**Figure 11C**), a 2.9 ± 0.1-fold induction of *IFITM1* mRNA (**Figure 11D**) and a robust 186-fold 50-fold induction of *MX1* (**Figure 11E**). Interpreting the KD and physiological expression experiments together, these experiments provide strong evidence that IRF1 is a direct transactivator of type I and III IFNs and ISGs in epithelial cells.

**Figure 12:**
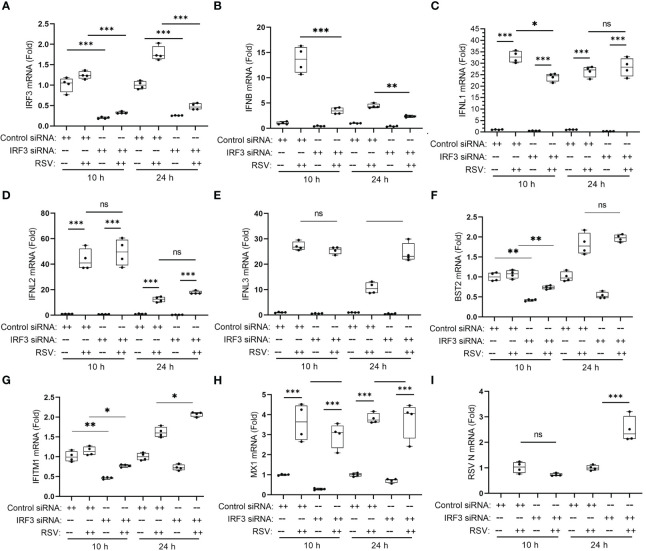
IRF3 mediates type I, but not type III IFNs in RSV-induced anti-viral response. hSAECs were transfected overnight by control or human IRF3-specific siRNA (Dharmacon SMARTpool, 25 nM) using DharmaFECT 1 (2.0 μl per 24-well), followed by 48 h culture in fresh medium. The siRNA transfected cells were then mock- or RSV-infected for 10 or 24 h. Q-RT-PCR was performed for the mRNA levels of *IRF3* **(A)**, *IFNβ* **(B)**, *IFNL1* **(C)**, *IFNL2* **(D)**, *IFNL3* **(E)**, BST2 **(F)**, IFITM1 **(G)**, *MX1* **(H)** and RSV N **(I)**. Error bars are ± SD with four independent replicates. **, P<0.01; ***, P<0.001, *post-hoc* analysis. Note the significant reduction in RSV-induced IFNβ in IRF3-silenced cells, the lack of effect on *IFL2, 3* mRNAs and the promoting effect of IRF3 knockdown on RSV replication in 24 h. n.s., not significant; *, P<0.05, *post-hoc* analysis.

IRF1 is a member of a family of inducible transcription factors whose expression co-regulate the type I IFN response, mediate oncogenic transformation and activate adaptive immunity (55). Of these family members, our work has shown that the IRF3 transcription factor has also been implicated in as an auxiliary transcription factor mediating the coordinated ISG response RSV in human airway epithelial cells (10, 17, 34). To better understand the contribution of IRF3 in the IRE1-XBP1s induction of ISGs, we conducted a series of experiments depleting IRF3 using siRNA. hSAECs were transfected with IRF3 siRNA or scrambled siRNA and infected for various times (10, 24 h) with RSV (MOI=1). Consistent with earlier findings, *IRF3* mRNA was not induced after 10 h of RSV infection and only increased by 1.7 ± 0.7-fold increase after 24 h of infection. In these experiments, IRF3 was effectively silenced at all time points measured, where *IRF3* mRNA was 20 ± 2% in mock-infected IRF3 KD cells *vs* scrambled transfectants at 10 h, and 26 ± 0.5% at 24 h (**Figure 12A**).

We further examined the effects of IRF3 KD on type I and III IFNs. Here we noted that the robust 13.6 ± 2.6-fold increase in IFNβ expression in control transfectants at 10 h was reduced to 3.6 ± 0.6-fold in the IRF3 KDs (**Figure 12B**). And similarly, IRF3 KD reduced IFNβ expression at the 24 h time point (**Figure 12B**).

In striking contrast, IRF3 KD had an initial effect on the type III IFN, *IFNL1* after 10 h of infection, but did not affect its later expression at 24 h (**Figure 12C**). There was no detectable effect on *IFNL2* and *IFNL3* expression either at the 10 h or 24 h of RSV infection (**Figures 12D, E**). IRF3 KD reduced the early response of *BST2* and *IFITM1* mRNAs, but did not affect the 24 h time points (**Figures 12F, G**) and had no detectable effect on the *MX1* gene (**Figure 12H**). IRF3 KD interfered with cellular anti-viral response to RSV as shown by the 2.5-fold increase in RSV replication after 24 h (**Figure 12I**). These findings indicate that IRF3 plays an important anti-viral role mediating primarily IFNβ expression and the early response of the ISGs, *BST2* and *IFITM1*, but does not play a significant role in type III *IFNL-1,-2* or *-3* mRNA response or late responses of *MX1*, *BST2* or *IFITM1*. We conclude the type III IFN genes are primarily driven by the IRE1α-XBP1s-IRF1 pathway, consistent with the RNA seq studies of IRF1-deficient epithelial cells (56).

### 3.9 Validation of IRE1α-XBP1s in RSV-induced IRF1 expression

**Figure 13:**
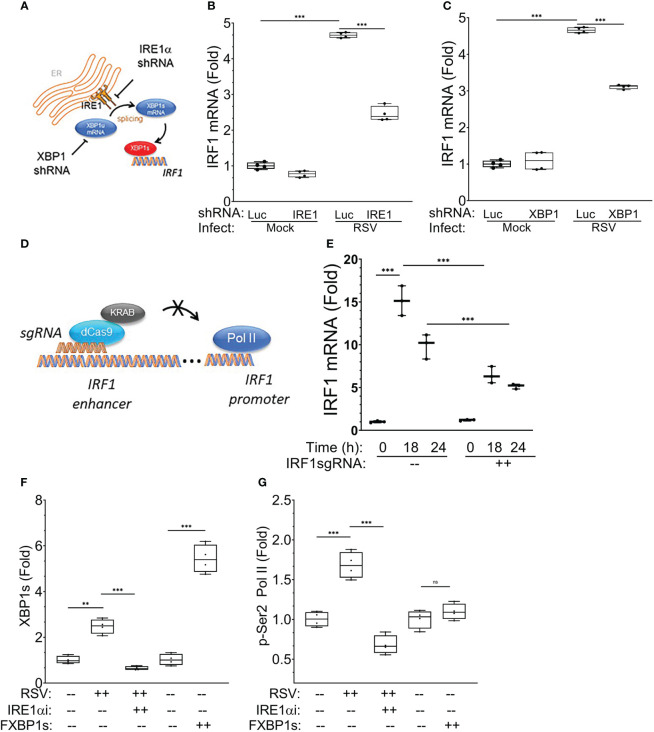
IRE1-XBP1s requirement in RSV-induced IRF1 expression. The requirement for IRE1α-XBP1s in *IRF1* mRNA expression was examined in hSAECs after lentiviral silencing. **(A)** Experimental schematic. **(B)** Effect of IRE1α silencing. Nontargeting shRNA and IRE1α-targeted shRNA expressing hSAECs were RSV infected for 24 h (MOI=1) and *IRF1* gene expression determined by Q-RT-PCR. Shown are 10-90% interquartile ranges of mRNA fold change relative to mock infected hSAECs. **(C)** Effect of XBP1 silencing. Nontargeting and XBP1-targeting lentivirus shRNA transduced cells were infected with RSV and IRF1 mRNA measured as above. ***, P<0.001, *post-hoc* analysis. **(D)** Role of IRF1 enhancer in RSV-induced IRF1 expression. Experimental schematic. hSAECs expressing NH2-terminal fusion of Krox KRAB repressor domain with enzymatically inactive Cas9 (dCas9) were transduced with nontargeting or *IRF1* 5’-enhancer-targeting single guide RNA (sgRNAs). The *IRF1*-specific sgRNA guides KRAB-dCas9 to the *IRF1* 5’-enhancer for transcriptional repression. **(E)** Effect of KRAB-dCas9 on RSV-induced IRF1 mRNA expression. KRAB-dCas9 expressing hSAECs were transduced with non-targeting or *IRF1* 5’-enhancer-targeting sgRNAs. Cells were RSV infected and IRF1 mRNA measured at 0, 18 and 24 h after infection. ***, P<0.001, *post-hoc* analysis. **(F, G)** XBP1s primes phospho-Ser2 Pol II binding to the *IRF1* enhancer. XChIP for XBP1s and p-Ser2 Pol II binding on the *IRF1* 5’-enhancer. hSAECs were mock- or RSV infected (MOI=1, 24 h) in the absence or presence of KIRA8, or transduced with empty lentiviral vector or FXBP1s for 48 h. For each XChIP, Q-gPCR for *IRF1* 5’-enhancer abundance was performed. Data are presented as fold change over mock-infected cells. Individual symbols are biological replicates. **(F)** XBP1s binding; **(G)** p-Ser2 Pol II binding. Error bars are ± SD. **, P<0.01, *post-hoc* analysis.

We sought to provide direct, independent evidence that the IRE1α-XBP1s pathway was required for *IRF1* mRNA expression. IRE1α and XBP1 were depleted separately using shRNA using an established knockdown system where RSV-induced *XBP1s* mRNA was reduced by ~60% and ~85%, respectively (19). In the presence of RSV infection, both *IRE1α* and *XBP1* silencing significantly reduced the expression of *IRF1* mRNA (by ~50% and ~45%, respectively) (**Figures 13A-C**). These data indicate that XBP1s contributes to RSV-inducible IRF1 expression.

### 3.10 Functional activity of the XBP1s binding site in the IRF1 enhancer

Our CUT&RUN data demonstrates that XBP1s binds to a putative regulatory element in the 5’ flanking sequence of the IRF1 gene whose fragmentation pattern is significantly modified by RSV infection (see **Figure 9**). To determine whether the XBP1s-binding enhancer is functionally required for inducible IRF1 expression, we tested the effect of targeting the enhancer sequence with a potent Krüppel-associated box (KRAB) repressor domain (38, 57). In this experiment, hSAECs expressing KRAB-dCas9 were transduced with non-targeting or *IRF1* 5’ enhancer-targeting single guide RNA (sgRNA; **Figure 13D**). Cells were then infected with RSV for 0, 18 and 24 h and expression of *IRF1* mRNA determined by Q-RT-PCR. We observed that the 15.2 ± 1.7-fold induction of *IRF1* mRNA at 18 h was significantly reduced to 6.4 ± 1-fold in the presence of IRF1 enhancer targeting sgRNA (P<0.0001, *post-hoc* t test, **Figure 13E**). Similarly, the 10 ± 1.4-fold increase of *IRF1* mRNA at 24 h was reduced to 5.2 ± 0.3-fold in the presence of *IRF1*-specific sgRNA (p<0.001). We interpret these data to indicate that the XBP1s binding enhancer element is functionally required for RSV-induced IRF1 activation.

### 3.11 XBP1s mediates phospho-Ser 2 Pol II recruitment to the IRF1 enhancer in the setting of RSV infection

We next conducted chromatin immunoprecipitation (XChIP) experiments of the *IRF1* enhancer in the absence or presence of RSV infection, or after FXBP1s transduction. Here we observed that RSV infection enhanced XBP1s binding to the *IRF1* enhancer. Additionally, XBP1s binding was significantly reduced by the treatment with the IRE1α inhibitor (**Figure 13F**). Separately, transduction with the FXBP1s-expressing lentivirus produced 5-fold increase in XBP1s binding over that of empty vector controls (**Figure 13F**). These data directly confirm that XBP1s interacts with *IRF1* enhancer, as suggested by the CUT&RUN binding peaks (cf **Figures 13F**, **Figure 9**).

XChIP experiments were next conducted for phospho-Ser 2 RNA Pol II (p-Ser2 Pol II) binding to the IRF1 enhancer. We found that RSV infection induced a 1.6-fold increase of p-Ser2 Pol II binding that was completely inhibited by IRE1α inhibition (**Figure 13G**). Strikingly, despite the strong induction of FXBP1s binding, FXBP1s transduction alone did not result in recruitment of p-Ser2 Pol II to the IRF1 enhancer. These data indicate to us that XBP1s binding is necessary, but not sufficient, for p-Ser2 Pol II recruitment to the IRF1 enhancer.

## 4 Discussion

RSV is a major human pathogen that causes hospitalizations in young children and is associated with a 2-fold increased risk of premature death from respiratory causes in adults (3). Because RSV replicates to high levels in lower airway epithelial cells, where the timing and character of the innate immune response mediates the pathogenesis of disease and resolution, understanding the innate response in small airway bronchiolar cells is important to understanding the pathogenesis of disease. In epithelial cells, RSV replicates in ER-proximal stress granules where it activates innate pathways controlled by kinase complexes activating MAPK, IKK and TBK1 that exhibit significant cross-talk. In addition to these well-established innate pathways, our studies have begun to elucidate the important role of the cellular UPR in the viral stress response. Here, RSV activation of the highly evolutionarily conserved IRE1-XBP1s arm of the UPR pathway activates hexosamine biosynthesis to reduce proteotoxicity, induces cellular plasticity and activates type I (IFNβ) and III (IFNL) IFN expression and hence represents the focus of this study. Type I/III IFNs are epithelial-secreted proteins that activate ~300 ISGs that play complex roles in limiting RSV infection and spread through autocrine and paracrine mechanisms to induce an anti-viral response, regulate translation and control apoptosis (58, 59).

In this study, we integrated RNA-seq and CUT&RUN analyses to provide insights into gene regulatory networks directly activated by the IRE1α-XBP1s pathway. Strikingly, although RSV activates ISG expression in a manner sensitive to IRE1αi treatment, few ISGs are directly bound by XBP1s. Instead, we are able to identify a subset of core cytokine regulators that play important regulatory functions in major signaling pathways important in RSV-induced transcriptional response, including the MAP kinase, IKK-RelA and TBK1-IFN signaling pathways. Here we focus on the activation and downstream role of IRF1 on type I/III IFNs and ISGs. Additional studies will be required to dissect the role of this group completely, but we are able to demonstrate the critical role of XBP1s-IRF1 signaling in RSV activation of type I and III IFNs and ISGs.

Our study identifies IRF1 as a key upstream mediator of the epithelial anti-viral ISG response, supported by the extensive IRF1 depletion and physiological overexpression experiments. IRF1 is one of the highly inducible IRF family members in epithelial cells, whose expression is triggered by IFN-independent and IFN-dependent mechanisms. Our earlier mechanistic studies have found that one of the potent IFN-independent, IRF1 activating pathways was mediated by an inducible NFκB-bromodomain containing 4 (BRD4) complex. Here, RSV replication triggers the IκB kinase (IKK) to releasing phosphorylated RelA from sequestered cytoplasmic stores (51). pRelA is coupled to p300/pCAF acetylation, promoting Lys310 Ac-RelA to form a complex with the positive transcription elongation factor, BRD4/CDK9. The RelA BRD4 CDK9, in turn, complex binds to the proximal IRF1 promoter, triggering phospho-Ser 2 carboxy-terminal domain (CTD) RNA polymerase (Pol) II formation to express IRF1 via a transcriptional elongation pathway.

The data in this study adds to the complex mode of IRF1 regulation that involves activation by the IRE1α-XBP1s pathway through a key transcriptional control element in the 5’ *IRF1* enhancer. Our findings extend this observation to demonstrate that XBP1s is a component of RSV-inducible enhancer controlling IRF1 expression, necessary for recruitment of p-Ser2 Pol II. This sequence is enriched in AP1 and RELA sequences, whose cognate transcription factors are also activated by RSV infection. This region is therefore a complex regulatory enhancer binding a myriad of inducible transcription factors.

Using sgRNA-guided site-specific targeting of a potent KRAB repressor domain to the chromatin, we are the first to demonstrate that the XBP1s-binding 5’-enhancer element of *IRF1* is functionally required for viral inducible activation of IRF1 expression. Interestingly, although XBP1s binds to the *IRF1* enhancer either in the absence or presence of RSV infection (**Figures 8**, **12**), ectopic XBP1s alone is incapable of recruiting the activated p-Ser2 Pol II as does RSV-induced IRE1-XBP1s. These chromatin immunoprecipitation experiments suggest that XBP1s is necessary, but not sufficient alone, for RSV-induced recruitment of activated phospho-Ser2 Pol II to the IRF1 gene. This phenomenon may suggest that the UPR functions to “prime” the IFN pathway for robust activation by incorporating a second signal generated by the PRRs.

Previous work in macrophages have shown that XBP1s alone does not activate robust IFN production, but, instead, potentiates IFN expression signaling downstream of TLR3 and TLR4 ligands (26). Our finding that XBP1s binds to the IRF1 5’ enhancer, producing enhanced cleavage, yet these cleavage patterns are enhanced by RSV replication suggests that XBP1s binding is modified by RSV signaling for full activation of the IRF1 gene. Further understanding of the nature of the RSV-assembled complexes will require significant advances in chromatin proteomics of the XBP1s-containing domains.

DNA binding and PCR selection studies seeking to define XBP1s binding motifs suggests that XBP1s binds to highly pleiotropic DNA sequences, and that these sequence preferences are affected by extracellular stimulus, cell-type and differentiation state. Our experimental design allows us to isolate the effect of innate stimulus on XBP1s binding patterns, where we find some ~ 786 XBP1s binding sites are affected by RSV infection versus mock-infected cells. We note that these sequences are enriched in AP-1 and Rel motifs, pathways known to be activated by RSV replication. We speculate that this phenomenon may be a consequence of RSV inducing expression of bZIP proteins that could function as XBP1s heterodimeric partners, such as ATF6 or NFY, affecting sequence recognition (48, 60). In this regard, we note that XBP1s binding to an AP-1/CRE element has been reported in the regulation of brain natriuretic peptide in cardiomyocytes (61). Whether this binding complex was an XBP1s homodimer or a heterodimeric XBP1s-AP1 complex was not determined. We interpret the enrichment of AP-1/RelA sequences to suggest that XBP1s cooperates with multiple other RSV inducible cis regulatory elements in the formation of a functional *IRF1* upstream enhancer, likely a transactivating ‘hub’ domain (62). More work will be required to understand how this complex regulatory element affects other RSV-inducible genes.

Alternatively, the RSV-induced modification of the XBP1 binding on the *IRF1* enhancer may be the consequence of XBP1s complexing with non-DNA binding chromatin regulatory proteins. The innate signaling pathways activated by RSV show extensive cross-talk with interactions between UPR with MAPK, TBK1 and IKK signaling complexes. Of significance, we recently found that IKKβ directly complexes with and phosphorylates XBP1s in response to TGFβ stimulation (63). Whether XBP1s-IKKβ complex is formed in response to RSV infection or interacts with the *IRF1* 5’-enhancer is an exciting question and will be the focus of future investigation.

Once induced, IRF1 mediates a dramatic ISG amplification, activating IRF transcription factors, IFNs and PRRs (10, 17, 64). With upregulation of the RIG-I and TLR PRRs, additional molecular patterns formed by RSV replicating in the cytoplasmic stress granule further amplify the ISG response. This IFN positive-feedback loop enables a robust response to replicating virus, and their secretion primes neighboring epithelial cells to elicit a protective antiviral state, commit to apoptosis, or activate macrophages, NK cells, and DCs that play important roles in adaptive immunity. Earlier work has identified the presence of STAT- and IRF1 binding enhancers that serve as a transcription factor “hub” important in IFNα-and LPS-responsive gene clusters (62). These data suggest that the XBP1s-catalyzed IRF1 expression may induce substantial genomic structural rearrangement to promote anti-viral immunity. The role of IRF1 in chromatin looping and formation of transcription factor “hubs” will be subjects of future investigation.

The actions of IRF1 on type I and III IFNs and ISG expression are further underscored by experiments employing silencing IRF1 and physiological expression of IRF1. Both of these experiments converge on the conclusion that IRF1 is functionally a direct transactivator of ISGs in epithelial cells, further strengthening the conclusions of this study. Our previous work examining the role of IRF1 clearly demonstrates that IRF1 is responsible for substantial anti-viral protection from RSV and RV infection in the type III IFN (34). Finally, our findings from IRF3 silencing show that IRF3 has little effect on type III IFN expression indicates the IRF3 and IRF1 pathways are complementary to a full antiviral response.

In summary, we advance the understanding of the IRE1α-XBP1s pathway in activation of the IIR. We demonstrate XBP1s regulates the expression of a group of cytokine regulators, including IRF1 through a 5’ enhancer. Using chromatin IP and genome-targeting KRAB repressor domain, the IRF1 enhancer and its binding by XBP1s are shown to be required for IRF1 expression, providing novel insights into how the UPR mediates inflammation and anti-viral activity in response to RSV infection in the small airways.

## Data availability statement

The datasets presented in this study can be found in online repositories. The names of the repository/repositories and accession number(s) can be found below: https://www.ncbi.nlm.nih.gov/genbank/, GSE228327 https://www.ncbi.nlm.nih.gov/genbank/, GSE228328.

## Author contributions

Conceptualization: DQ and AB. Methodology: DQ, XX, JY and AB. Investigation: DQ, XX, YZ, JY and AB. Data curation: DQ, XX, YZ, JY and AB. Writing—original draft preparation: DQ and AB. Writing—review and editing: DQ, XX, JY and AB. Funding acquisition: DQ, JY and AB. All authors have read and agreed to the published version of the manuscript.

## References

[1] Stockman LJ Curns AT Anderson LJ Fischer-Langley G. Respiratory syncytial virus-associated hospitalizations among infants and young children in the United States, 1997-2006. Pediatr Infect Dis J (2012) 31:5–9. doi: 10.1097/INF.0b013e31822e68e6 21817948

[2] Shi T McAllister DA O'Brien KL Simoes EAF Madhi SA Gessner BD. Global, regional, and national disease burden estimates of acute lower respiratory infections due to respiratory syncytial virus in young children in 2015: a systematic review and modelling study. Lancet (2017) 390:946–58. doi: 10.1016/S0140-6736(17)30938-8 PMC5592248 28689664

[3] Allinson JP Chaturvedi N Wong A Shah I Donaldson GC Wedzicha JA. Early childhood lower respiratory tract infection and premature adult death from respiratory disease in Great Britain: a national birth cohort study. Lancet (2023) 401:1183–3. doi. doi: 10.1016/S0140-6736(23)00131-9 36898396

[4] Fauroux B Simoes EAF Checchia PA Paes B Figueras-Aloy J Manzoni P. The burden and long-term respiratory morbidity associated with respiratory syncytial virus infection in early childhood. Infect Dis Ther (2017) 6:173–97. doi: 10.1007/s40121-017-0151-4 PMC5446364 28357706

[5] Mosscrop LG Williams TC Tregoning JS. Respiratory syncytial virus after the SARS-CoV-2 pandemic — what next? Nat Rev Immunol (2022) 22:589–90. doi: 10.1038/s41577-022-00764-7 PMC9281204 35831610

[6] Johnson JE Gonzales RA Olson SJ Wright PF Graham BS. The histopathology of fatal untreated human respiratory syncytial virus infection. Mod Pathol (2007) 20:108–19. doi: 10.1038/modpathol.3800725 17143259

[7] Jozwik A Habibi MS Paras A Zhu J Guvenel A Dhariwal J. RSV-specific airway resident memory CD8+ T cells and differential disease severity after experimental human infection. Nat Commun (2015) 6:10224. doi: 10.1038/ncomms10224 26687547 PMC4703893

[8] Zhang Y Luxon BA Casola A Garofalo RP Jamaluddin M Brasier AR. Expression of respiratory syncytial virus-induced chemokine gene networks in lower airway epithelial cells revealed by cDNA microarrays. J Virol (2001) 75:9044–58. doi: 10.1128/jvi.75.19.9044-9058.2001 PMC114473 11533168

[9] Tian B Zhang Y Luxon BA Garofalo RP Casola A Sinha M. Identification of NF-kappaB-dependent gene networks in respiratory syncytial virus-infected cells. J Virol (2002) 76:6800–14. doi: 10.1128/jvi.76.13.6800-6814.2002 PMC136270 12050393

[10] Liu P Jamaluddin M Li K Garofalo RP Casola A Brasier AR. Retinoic acid-inducible gene I mediates early antiviral response and Toll-like receptor 3 expression in respiratory syncytial virus-infected airway epithelial cells. J Virol (2007) 81:1401–11. doi: 10.1128/JVI.01740-06 PMC1797494 17108032

[11] Hosakote YM Brasier AR Casola A Garofalo RP Kurosky A. Respiratory syncytial virus infection triggers epithelial HMGB1 release as a damage-associated molecular pattern promoting a monocytic inflammatory response. J Virol (2016) 90:9618–31. doi: 10.1128/JVI.01279-16 PMC5068515 27535058

[12] Zhao Y Jamaluddin M Zhang Y Sun H Ivanciuc T Garofalo RP. Systematic analysis of cell-type differences in the epithelial secretome reveals insights into the pathogenesis of respiratory syncytial virus-induced lower respiratory tract infections. J Immunol (2017) 198:3345–64. doi: 10.4049/jimmunol.1601291 PMC5380581 28258195

[13] Tian B Yang J Zhao Y Ivanciuc T Sun H Wakamiya M. Central role of the NF-kappaB pathway in the scgb1a1-expressing epithelium in mediating respiratory syncytial virus-induced airway inflammation. J Virol (2018) 92(11):e00441–18. doi: 10.1128/JVI.00441-18 PMC5952137 29593031

[14] Brownsword MJ Locker N. A little less aggregation a little more replication: Viral manipulation of stress granules. WIREs RNA (2023) 14:e1741. doi: 10.1002/wrna.1741 35709333 PMC10078398

[15] Lindquist ME Lifland AW Utley TJ Santangelo PJ Crowe JE Jr. Respiratory syncytial virus induces host RNA stress granules to facilitate viral replication. J Virol (2010) 84:12274–84. doi: 10.1128/jvi.00260-10 PMC2976418 20844027

[16] Seth RB Sun L Ea C-K Chen ZJ. Identification and characterization of MAVS, a mitochondrial antiviral signaling protein that activates NF-κB and IRF3. Cell (2005) 122:669–82. doi: 10.1016/j.cell.2005.08.012 16125763

[17] Liu P Lu M Tian B Li K Garofalo RP Prusak D. Expression of an IKKgamma splice variant determines IRF3 and canonical NF-kappaB pathway utilization in ssRNA virus infection. PloS One (2009) 4:e8079. doi: 10.1371/journal.pone.0008079 19956647 PMC2778955

[18] Brasier AR Qiao D Zhao Y. The hexosamine biosynthetic pathway links innate inflammation with epithelial-mesenchymal plasticity in airway remodeling. Front Pharmacol (2021) 12:808735. doi: 10.3389/fphar.2021.808735 35002741 PMC8727908

[19] Qiao D Skibba M Xu X Garofalo RP Zhao Y Brasier AR. Paramyxovirus replication induces the hexosamine biosynthetic pathway and mesenchymal transition via the IRE1α-XBP1s arm of the unfolded protein response. Am J Physiol Lung Cell Mol Physiol (2021) 321:L576–94. doi: 10.1152/ajplung.00127.2021 PMC8461800 34318710

[20] Grootjans J Kaser A Kaufman RJ Blumberg RS. The unfolded protein response in immunity and inflammation. Nat Rev Immunol (2016) 16:469–84. doi: 10.1038/nri.2016.62 PMC5310224 27346803

[21] Anderson K Stott EJ Wertz GW. Intracellular processing of the human respiratory syncytial virus fusion glycoprotein: amino acid substitutions affecting folding, transport and cleavage. J Gen Virol (1992) 73(Pt 5):1177–88. doi: 10.1099/0022-1317-73-5-1177 1375280

[22] Zhao Y Qiao D Skibba M Brasier AR. The IRE1α-XBP1s arm of the unfolded protein response activates N-glycosylation to remodel the subepithelial basement membrane in paramyxovirus infection. Int J Mol Sci (2022) 23:9000. doi: 10.3390/ijms23169000 36012265 PMC9408905

[23] Xu X Qiao D Dong C Mann M Garofalo RP Keles S. The SWI/SNF-related, matrix associated, actin-dependent regulator of chromatin A4 core complex represses respiratory syncytial virus-induced syncytia formation and subepithelial myofibroblast transition. Front Immunol (2021) 12:633654. doi: 10.3389/fimmu.2021.633654 33732255 PMC7957062

[24] Gilardini Montani MS Falcinelli L Santarelli R Granato M Romeo MA Cecere N. KSHV infection skews macrophage polarisation towards M2-like/TAM and activates Ire1 α-XBP1 axis up-regulating pro-tumorigenic cytokine release and PD-L1 expression. Br J Cancer (2020) 123:298–306. doi: 10.1038/s41416-020-0872-0 32418990 PMC7374093

[25] Eckard SC Rice GI Fabre A Badens C Gray EE Hartley JL. The SKIV2L RNA exosome limits activation of the RIG-I-like receptors. Nat Immunol (2014) 15:839–45. doi: 10.1038/ni.2948 PMC4139417 25064072

[26] Smith JA Turner MJ DeLay ML Klenk EI Sowders DP Colbert RA. Endoplasmic reticulum stress and the unfolded protein response are linked to synergistic IFN-beta induction via X-box binding protein 1. Eur J Immunol (2008) 38:1194–203. doi: 10.1002/eji.200737882 PMC2838478 18412159

[27] Zeng L Liu YP Sha H Chen H Qi L Smith JA. XBP-1 couples endoplasmic reticulum stress to augmented IFN-beta induction via1 a cis-acting enhancer in macrophages. J Immunol (2010) 185:2324–30. doi: 10.4049/jimmunol.0903052 PMC2916979 20660350

[28] Qiao D Skibba M Xu X Brasier AR. Genomic targets of the IRE1-XBP1s pathway in mediating metabolic adaptation in epithelial plasticity. Nucleic Acids Res (2023) 51(8):3650–70. doi: 10.1093/nar/gkad077 PMC10164557 36772828

[29] Acosta-Alvear D Zhou Y Blais A Tsikitis M Lents NH Arias C. XBP1 controls diverse cell type- and condition-specific transcriptional regulatory networks. Mol Cell (2007) 27:53–66. doi: 10.1016/j.molcel.2007.06.011 17612490

[30] Pramanik J Chen X Kar G Henriksson J Gomes T Park J-E. Genome-wide analyses reveal the IRE1α-XBP1 pathway promotes T helper cell differentiation by resolving secretory stress and accelerating proliferation. Genome Med (2018) 10:76. doi: 10.1186/s13073-018-0589-3 30355343 PMC6199730

[31] Thamsen M Ghosh R Auyeung VC Brumwell A Chapman HA Backes BJ. Small molecule inhibition of IRE1α kinase/RNase has anti-fibrotic effects in the lung. PloS One (2019) 14:e0209824. doi: 10.1371/journal.pone.0209824 30625178 PMC6326459

[32] Patro R Duggal G Love MI Irizarry RA Kingsford C. Salmon provides fast and bias-aware quantification of transcript expression. Nat Methods (2017) 14:417–9. doi: 10.1038/nmeth.4197 PMC5600148 28263959

[33] Love MI Huber W Anders S. Moderated estimation of fold change and dispersion for RNA-seq data with DESeq2. Genome Biol (2014) 15:550. doi: 10.1186/s13059-014-0550-8 25516281 PMC4302049

[34] Yang J Tian B Sun H Garofalo RP Brasier AR. Epigenetic silencing of IRF1 dysregulates type III interferon responses to respiratory virus infection in epithelial to mesenchymal transition. Nat Microbiol (2017) 2:17086. doi: 10.1038/nmicrobiol.2017.86 28581456 PMC5501188

[35] Zhang Y Liu T Eeckhoute J Johnson DS Bernstein DE Nusbaum C. Model-based analysis of chIP-seq (MACS). Genome Biol (2008) 9:R137. doi: 10.1186/gb-2008-9-9-r137 18798982 PMC2592715

[36] Meers MP Tenenbaum D Henikoff S. Peak calling by Sparse Enrichment Analysis for CUT&RUN chromatin profiling. Epigenet Chromatin (2019) 12:42. doi: 10.1186/s13072-019-0287-4 PMC6624997 31300027

[37] Sanjana NE Shalem O Zhang F. Improved vectors and genome-wide libraries for CRISPR screening. Nat Methods (2014) 11:783–4. doi: 10.1038/nmeth.3047 PMC4486245 25075903

[38] Gilbert LA Horlbeck MA Adamson B Villalta JE Chen Y Whitehead EH. Genome-scale CRISPR-mediated control of gene repression and activation. Cell (2014) 159:647–61. doi: 10.1016/j.cell.2014.09.029 PMC4253859 25307932

[39] Nowak DE Tian B Brasier AR. Two-step cross-linking method for identification of NF-kappaB gene network by chromatin immunoprecipitation. Biotechniques (2005) 39:715–25. doi: 10.2144/000112014 16315372

[40] Tian B Yang J Brasier AR. Two-step cross-linking for analysis of protein-chromatin interactions. Methods Mol Biol (2012) 809:105–20. doi: 10.1007/978-1-61779-376-9_7 PMC4148016 22113271

[41] Stark R Brown G. DiffBind: Differential binding analysis of ChIP-Seq peak data. (2012). Available at: https://bioconductor.org/packages/devel/bioc/vignettes/DiffBind/inst/doc/DiffBind.pdf. (Accessed 7/2023).

[42] Heinz S Benner C Spann N Bertolino E Lin YC Laslo P. Simple combinations of lineage-determining transcription factors prime cis-regulatory elements required for macrophage and B cell identities. Mol Cell (2010) 38:576–89. doi: 10.1016/j.molcel.2010.05.004 PMC2898526 20513432

[43] Hassan I Gaines KS Hottel WJ Wishy RM Miller SE Powers LS. Inositol-requiring enzyme 1 inhibits respiratory syncytial virus replication. J Biol Chem (2014) 289:7537–46. doi: 10.1074/jbc.M113.510594 PMC3953267 24497642

[44] Tian B Li X Kalita M Widen SG Yang J Bhavnani SK. Analysis of the TGFbeta-induced program in primary airway epithelial cells shows essential role of NF-kappaB/RelA signaling network in type II epithelial mesenchymal transition. BMC Genomics (2015) 16:529. doi: 10.1186/s12864-015-1707-x 26187636 PMC4506436

[45] Tian B Widen SG Yang J Wood TG Kudlicki A Zhao Y. The NFkappaB subunit RELA is a master transcriptional regulator of the committed epithelial-mesenchymal transition in airway epithelial cells. J Biol Chem (2018) 293:16528–45. doi: 10.1074/jbc.RA118.003662 PMC6200927 30166344

[46] Zhang J Jamaluddin M Zhang Y Widen SG Sun H Brasier AR. Type II epithelial-mesenchymal transition upregulates protein N-glycosylation to maintain proteostasis and extracellular matrix production. J Proteome Res (2019) 18:3447–60. doi: 10.1021/acs.jproteome.9b00342 PMC7195216 31424945

[47] Xu X Mann M Qiao D Brasier AR. Alternative mRNA processing of innate response pathways in respiratory syncytial virus (RSV) infection. Viruses (2021) 13(2):218. doi: 10.3390/v13020218 33572560 PMC7912025

[48] Clauss IM Chu M Zhao JL Glimcher LH. The basic domain/leucine zipper protein hXBP-1 preferentially binds to and transactivates CRE-like sequences containing an ACGT core. Nucleic Acids Res (1996) 24:1855–64. doi: 10.1093/nar/24.10.1855 PMC145876 8657566

[49] McLean CY Bristor D Hiller M Clarke SL Schaar BT Lowe CB. GREAT improves functional interpretation of cis-regulatory regions. Nat Biotechnol (2010) 28:495–501. doi: 10.1038/nbt.1630 20436461 PMC4840234

[50] Yamane D Feng H Rivera-Serrano EE Selitsky SR Hirai-Yuki A Das A. Basal expression of interferon regulatory factor 1 drives intrinsic hepatocyte resistance to multiple RNA viruses. Nat Microbiol (2019) 4:1096–104. doi: 10.1038/s41564-019-0425-6 PMC6588457 30988429

[51] Garofalo R Sabry M Jamaluddin M Yu RK Casola A Ogra PL. Transcriptional activation of the interleukin-8 gene by respiratory syncytial virus infection in alveolar epithelial cells: nuclear translocation of the RelA transcription factor as a mechanism producing airway mucosal inflammation. J Virol (1996) 70:8773–81. doi: 10.1128/JVI.70.12.8773-8781.1996 PMC190974 8971006

[52] Manley GCA Parker LC Zhang Y. Emerging regulatory roles of dual-specificity phosphatases in inflammatory airway disease. Int J Mol Sci (2019) 20(3):678. doi: 10.3390/ijms20030678 30764493 PMC6387402

[53] Creyghton MP Cheng AW Welstead GG Kooistra T Carey BW Steine EJ. Histone H3K27ac separates active from poised enhancers and predicts developmental state. Proc Natl Acad Sci (2010) 107:21931–6. doi: 10.1073/pnas.1016071107 PMC3003124 21106759

[54] Kim PKM Armstrong M Liu Y Yan P Bucher B Zuckerbraun BS. IRF-1 expression induces apoptosis and inhibits tumor growth in mouse mammary cancer cells in *vitro* and in *vivo* . Oncogene (2004) 23:1125–35. doi: 10.1038/sj.onc.1207023 14762441

[55] Tamura T Yanai H Savitsky D Taniguchi T. The IRF family transcription factors in immunity and oncogenesis. Annu Rev Immunol (2008) 26:535–84. doi: 10.1146/annurev.immunol.26.021607.090400 18303999

[56] Panda D Gjinaj E Bachu M Squire E Novatt H Ozato K. IRF1 maintains optimal constitutive expression of antiviral genes and regulates the early antiviral response. Front Immunol (2019) 10:1019. doi: 10.3389/fimmu.2019.01019 31156620 PMC6529937

[57] Witzgall R O'Leary E Leaf A Onaldi D Bonventre JV. The Krüppel-associated box-A (KRAB-A) domain of zinc finger proteins mediates transcriptional repression. Proc Natl Acad Sci U.S.A. (1994) 91:4514–8. doi: 10.1073/pnas.91.10.4514 PMC43816 8183940

[58] Smieja J Jamaluddin M Brasier AR Kimmel M. Model-based analysis of interferon-beta induced signaling pathway. Bioinformatics (2008) 24:2363–9. doi: 10.1093/bioinformatics/btn400 PMC2720726 18713791

[59] Czerkies M Korwek Z Prus W Kochanczyk M Jaruszewicz-Blonska J Tudelska K. Cell fate in antiviral response arises in the crosstalk of IRF, NF-kappaB and JAK/STAT pathways. Nat Commun (2018) 9:493. doi: 10.1038/s41467-017-02640-8 29402958 PMC5799375

[60] Yamamoto K Yoshida H Kokame K Kaufman RJ Mori K. Differential contributions of ATF6 and XBP1 to the activation of endoplasmic reticulum stress-responsive cis-acting elements ERSE, UPRE and ERSE-II. J Biochem (2004) 136:343–50. doi: 10.1093/jb/mvh122 15598891

[61] Sawada T Minamino T Fu HY Asai M Okuda K Isomura T. X-box binding protein 1 regulates brain natriuretic peptide through a novel AP1/CRE-like element in cardiomyocytes. J Mol Cell Cardiol (2010) 48:1280–9. doi: 10.1016/j.yjmcc.2010.02.004 20170659

[62] Santiago-Algarra D Souaid C Singh H Dao LTM Hussain S Medina-Rivera A. Epromoters function as a hub to recruit key transcription factors required for the inflammatory response. Nat Commun (2021) 12:6660. doi: 10.1038/s41467-021-26861-0 34795220 PMC8602369

[63] Zhao Y Zhang J Sun H Brasier AR. Crosstalk of the ikappaB kinase with spliced X-box binding protein 1 couples inflammation with glucose metabolic reprogramming in epithelial-mesenchymal transition. J Proteome Res (2021) 20:3475–88. doi: 10.1021/acs.jproteome.1c00093 PMC9517173 34124911

[64] Tian B Yang J Zhao Y Ivanciuc T Sun H Garofalo RP. BRD4 couples NF-kappaB/relA with airway inflammation and the IRF-RIG-I amplification loop in respiratory syncytial virus infection. J Virol (2017) 91(6):e00007–17. doi: 10.1128/JVI.00007-17 PMC5331805 28077651

